# The Consistency of Prior Movements Shapes Locomotor Use-Dependent Learning

**DOI:** 10.1523/ENEURO.0265-20.2021

**Published:** 2021-09-08

**Authors:** Jonathan M. Wood, Susanne M. Morton, Hyosub E. Kim

**Affiliations:** 1 Physical Therapy Department, University of Delaware, Newark, DE 19713; 2 Graduate Program in Biomechanics and Movement Science, University of Delaware, Newark, DE 19713; 3 Department of Psychological and Brain Sciences, University of Delaware, Newark, DE 19713; 4 Department of Biomedical Engineering, University of Delaware, Newark, DE 19713

**Keywords:** computational modeling, locomotion, motor learning, motor variability, use-dependent learning

## Abstract

Repetition is an indispensable component of motor skill acquisition. However, it is unknown how consistent repeated movement patterns must be to engage an implicit “use-dependent” learning mechanism. In this Registered Report, we tackled this question through a combination of computational modeling, simulations, and behavioral experiments involving visually-guided treadmill walking. Our hypotheses were formalized by two distinct computational models: in the two-process Strategy plus Use-Dependent model, use-dependent learning is viewed as a slowly updating and slowly decaying bias in the direction of repeated movements. The Adaptive Bayesian model frames use-dependent learning as an emergent property of quickly adapting prior probabilities of target step lengths. Critically, the Adaptive Bayesian model is much more sensitive to variable practice than the Strategy plus Use-Dependent model. To test these hypotheses, human participants (*N* = 18, 10 females) learned a novel asymmetric stepping pattern under three conditions with differing amounts of practice consistency during a learning block. We probed use-dependent movement biases immediately postlearning by asking participants to “walk normally” during a washout block with no visual feedback (VF). We found that the total magnitude of use-dependent learning depended on practice consistency during learning, consistent with the Adaptive Bayesian model. However, this dependence faded quickly as biases became similar in magnitude over subsequent strides across all conditions, an observation more consistent with the Strategy plus Use-Dependent model. Simple *post hoc* adjustments to the Strategy plus Use-Dependent model made clear that these seemingly opposing effects of practice consistency can result from a unitary use-dependent learning process shaped by recent movement history.

## Significance Statement

Practice, in the form of movement repetition, is central to motor learning. However, we do not know how consistently a person must move to benefit from repeated practice. We aimed to answer this question through a combination of computational modeling and behavioral experiments. We found that the total magnitude of use-dependent learning was dependent on practice consistency, but this dependence faded quickly, leaving a small but sustained aftereffect that is resistant to completely washing out. A simple model that learned only from recent movement history could explain our results. These findings should have broad implications for the study of locomotor learning as repetition is central to both basic locomotor learning and gait rehabilitation studies.

## Introduction

Practice, in the form of movement repetition, is widely recognized as an indispensable component of motor skill acquisition (Schmidt and Lee, 2005). Even after acquiring a skill, repetition continues to play an important role. For example, repetition reduces the time required to prepare a movement (Wong et al., 2017; Mawase et al., 2018), increases movement speed (Hammerbeck et al., 2014), and biases future movements in the direction of the repeated movements, phenomena that are collectively referred to as “use-dependent learning” (Classen et al., 1998; Diedrichsen et al., 2010). The use-dependent biasing of movements may help explain why, for instance, a basketball player continues to practice her free throws years after she initially learned how to shoot, and even mimics those motions without the ball moments before shooting a free throw during a game. However, since no two movements can ever be identical, how consistent must the basketball player’s motions be during practice to benefit from use-dependent learning?

Most studies of use-dependent learning have examined the phenomenon during upper-extremity movements (Classen et al., 1998; Diedrichsen et al., 2010; Orban de Xivry et al., 2011; Verstynen and Sabes, 2011). The relatively sparse literature on use-dependent learning in locomotion is surprising, given the highly repetitive nature of walking. Locomotion is, by definition, the repetition of a cyclical movement pattern. Thus, the cyclical, repetitive nature of walking creates an excellent opportunity to study use-dependent learning in an ecologically valid context.

A recent study demonstrated that use-dependent learning explains step asymmetry aftereffects in visually guided treadmill walking (Wood et al., 2020), despite previous interpretations that aftereffects observed during similar paradigms were primarily because of learning from sensory prediction errors, i.e., sensorimotor adaptation (Kim and Krebs, 2012; Hussain et al., 2013; Kim and Mugisha, 2014; Statton et al., 2016; Cherry-Allen et al., 2018; French et al., 2018). In the study by Wood et al. (2020), visual targets were used to guide participants into walking with a step length asymmetry. Critically, for one of the experimental groups, all visual feedback (VF) was veridical, and participants were fully aware that they were being guided by the targets to practice walking asymmetrically. Therefore, the small but persistent aftereffects observed during washout, when all VF was removed and participants were instructed to “walk normally,” were highly consistent with use-dependent learning. As repetition of novel gait patterns is inherent to nearly all locomotor learning studies, these findings suggest that use-dependent learning may play an important yet underappreciated role in this body of literature. Thus, critical questions regarding use-dependent learning during locomotion remain. Given that movement is intrinsically variable, how consistent must the walking pattern be to engage use-dependent learning? Additionally, what are the computational principles underlying use-dependent learning in locomotion?

Here, through computational modeling, simulations, and a series of behavioral experiments, we directly tackled the question of how the consistency of movement patterns impacts use-dependent learning. Our competing hypotheses were formalized by two distinct computational models of how use-dependent learning may arise. In model 1, the Strategy plus Use-Dependent model, two learning processes act in parallel. A voluntary, strategic learning process that is active when the goal is to match step lengths to visual targets, and an automatic, slowly updating use-dependent learning process that biases movements in the direction of immediately preceding movements (Diedrichsen et al., 2010). Because of the slow learning and slow forgetting nature of use-dependent learning in this model, the use-dependent bias is robust to changes in movement consistency. In model 2, the Adaptive Bayesian model, adopted from a study of reaching (Verstynen and Sabes, 2011), use-dependent learning is framed as a process of combining quickly adapting prior probabilities of target (step) locations with current sensory estimates of where to step. Thus, in direct contrast to the Strategy plus Use-Dependent model, the magnitude of use-dependent biases is directly related to the consistency of the environment, or target locations. Concretely, the Adaptive Bayesian model predicts a progressive decrease in use-dependent bias magnitude with less consistent practice while the Strategy plus Use-Dependent model predicts similar use-dependent bias magnitude regardless of practice consistency.

Critically, while these two computational accounts provide putative explanations for use-dependent biases, they differ markedly in their theoretical underpinnings and, to our knowledge, have not been directly compared with each other. Therefore, we designed a set of walking experiments that systematically varied environmental consistency during learning and assessed the state of use-dependent biases during no-feedback washout trials to discriminate between these two competing theories on the underlying constraints of use-dependent learning.

## Materials and Methods

This study was conducted as a Registered Report. First, we developed and ran simulations of the Strategy plus Use-Dependent and Adaptive Bayesian models to characterize the distinct predictions these two models make with regard to use-dependent biases. Next, we collected pilot data and performed model recovery analysis to ensure feasibility of both our behavioral methods as well as our model fitting procedures. We then submitted the Registered Report proposal and received in-principal acceptance on October 23, 2020. We publicly posted the preregistered report on the Open Science Framework (https://osf.io/qfw9z) and soon after, initiated data collection and analysis. We provide all data and analysis scripts as well as our laboratory log at https://osf.io/49mxu.

### Behavioral methods

#### Participants

Young, healthy male and female individuals between the ages of 18–40 years were recruited to participate in this study. Participants were included if they were naive to locomotor learning tasks. Participants were excluded if they had a history of any neurologic, psychiatric or cognitive conditions, or had any cardiovascular or musculoskeletal problems that limited their walking. This study was approved by the University of Delaware institutional review board, and all participants provided written informed consent before participation.

#### Paradigm

**Figure 1.**
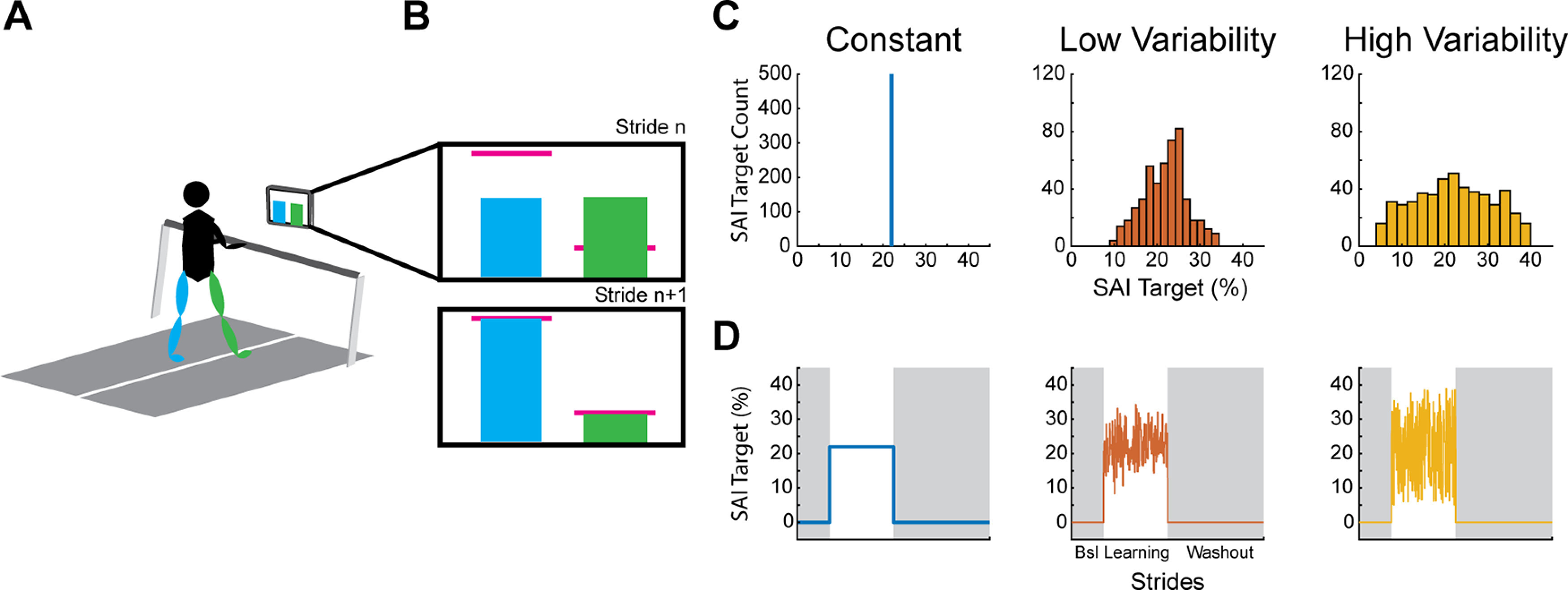
Experimental setup. ***A***, Participants walked on a treadmill while watching feedback of their step lengths. Their step lengths were represented as a blue (left) and green (right) bar which increased in height during the swing phase and held on the screen at the moment of heel strike. ***B***, During the learning phase, the participants aimed for the pink horizontal target lines which were derived from their baseline step lengths. On the first stride of learning the target was offset from their baseline (***B***, top panel), and the subject had to adjust their step length on subsequent strides to hit the target (***B***, bottom panel). ***C***, Example target distribution for each condition. During the constant condition, targets were always 22% SAI during the learning phase. During the low variability condition, targets were drawn from a normal distribution centered around 22% step asymmetry index (SAI; see equation 1) and a SD of 5% SAI. During the high variability condition, targets were drawn from a uniform distribution between 5% and 39% SAI. Note the different scales for the *y*-axes. ***D***, Learning schedule for each condition. Each condition included a baseline (Bsl), learning, and washout phase. Shaded regions indicate no VF was shown on the screen and participants were told to “walk normally,” so the target is effectively 0% SAI. During the learning phase, targets varied based on the condition.

Participants performed three sessions of walking spaced 5–10 d apart. During each session, they walked on a dual belt treadmill (with the belts tied throughout the experiment) at a speed between 1.0 and 1.2 m/s, selected by the participants to ensure a comfortable walking speed based on their anthropometrics. Participants wore a ceiling mounted harness, which did not provide any body weight support, and held onto a handrail for safety during all walking phases. A computer monitor placed 60 cm in front of the treadmill provided real-time VF of the participant’s step lengths (Fig. 1*A*; The Motion Monitor Toolbox, Innovative Sports Training Inc.).

The VF was in the form of two bar graphs with a blue bar representing the left leg’s step length and a green bar representing the right leg’s step length (Fig. 1*B*). The bars were time synchronized with each respective limb’s swing phase, increasing in height until the limb reached heel strike at which point the bar was held on the screen until the next swing phase began. There was also a pink horizontal target line for each leg which was derived from each participant’s baseline step length for each session and served as the target during that session’s learning phase.

Each of the three sessions of walking involved a similar block schedule. Participants were first told to “look forward and walk normally” on the treadmill during the baseline phase for 250 strides (i.e., 250 consecutive left heel strikes). No VF was presented on the monitor during the baseline phase. In order for participants to understand how changing each step length changed the height of the bars on the screen, they performed a short (25 strides) Orientation phase following Baseline during the first session only. During orientation, the participants were guided in changing their step lengths (green and blue bars) relative to their baseline (pink horizontal target lines, one for each leg). During the learning phase, participants were told to hit the pink horizontal target lines exactly with each step for 500 strides. Both target lines were changed relative to their baseline step length, leading the participants to take a longer step with the left leg and a shorter step with the right leg. The target lines had a width of ±2 % step length change from baseline. The researcher provided participants with a prompt to continue hitting the target lines every 100 strides during the learning phase. During the washout phase, the feedback was removed from the screen and participants were told to look forward and walk normally for 750 strides. The treadmill was stopped briefly between each phase so that instructions could be provided for the next phase.

#### Conditions

We systematically manipulated the independent variable, the consistency of target positions, during the learning phase. To accomplish this, participants completed three different conditions. (1) In the constant condition, the target locations were set to a constant 22% step asymmetry throughout the learning phase. (2) In the low variability (LV) condition, target locations were drawn from a normal distribution with a mean of 22% and SD of 5%. And (3), in the high variability (HV) condition, the targets were drawn from a uniform distribution with a range of 5−39% step asymmetry (Fig. 1*C*,*D*). Thus, all three conditions had a nearly identical average step asymmetry target of 22% (small discrepancies in the variable conditions because of drawing random samples), but the target variability for each condition was markedly different. This study design was intended to isolate the effects of target consistency on the use-dependent bias during washout. Based on our pilot testing, changing the target on a stride-by-stride basis made the task too difficult for participants; thus, for both the low variability and high variability conditions, targets changed, with equal probability, every one to five strides. To prevent contamination from potential order effects, we counterbalanced the order of conditions across all participants.

#### Data collection

Kinematic data were collected at a frequency of 100 Hz using a Vicon MX40 motion capture system with eight cameras and Nexus software (Vicon Motion Systems). We used a custom marker set with 11 total retroreflective markers, one for each greater trochanter, lateral knee, heel, lateral malleolus, and fifth metatarsal head. The eleventh marker was placed on the left first metatarsal head to ensure the tracking system could differentiate between the right and left feet.

#### Analysis pipeline

First, any gaps in the kinematic data were filled using a Woltring filter for small gaps (one to four frames) and Pattern Fill for larger gaps (more than four frames) in Nexus. The remainder of the data analysis were performed with custom-written MATLAB scripts (The MathWorks). The code/software described in the paper is freely available online at https://osf.io/49mxu. The code is available as Extended Data. Kinematic data were low pass filtered at 10 Hz using a fourth order Butterworth filter. Kinematic marker data were used to select *heel strike* when the heel marker velocity transitioned from positive to negative and *toe off* when the fifth metatarsal head marker velocity transitioned from negative to positive (Zeni et al., 2008). Step lengths were calculated as the sagittal difference between the leading and trailing heel markers at the moment of leading heel strike. The step length during the last 50 strides of the baseline phase was averaged and used to derive each legs’ respective target lines during that session’s learning phase. Step lengths were used to calculate our primary outcome, step asymmetry index (SAI):

(1) $$SAI=\frac{\left(Step\;Length_{LONG}-Step\;Length_{SHORT}\right)}{\left(Step\;Length_{LONG}+Step\;Length_{SHORT}\right)}*100\%$$

10.1523/ENEURO.0265-20.2021.ed Extended Data The Code. Download Extended Data, ZIP file.

Thus, SAI represents the difference between the two step lengths normalized by their sum. We express this measure as a percentage where 0% is perfect symmetry and SAIs further away from 0% indicate greater asymmetry. By convention, the SAI during learning was always positive. SAI was calculated on a stride-by-stride basis throughout all walking phases. We corrected for SAI baseline biases for each participant and each respective training session by subtracting the mean SAI of the last 50 strides of the baseline phase from all strides for that respective session. This baseline corrected measure was used for the remainder of our analyses.

We also calculated limb placement asymmetry. Leading limb foot placement was calculated as the sagittal distance between the hip and ankle marker during that limb’s heel strike and trailing limb placement was calculated as the sagittal distance between the same markers during that limb’s toe off. Leading and trailing limb placement asymmetry was calculated as the difference between the long and short leading and trailing limb placement, respectively (Finley et al., 2015; Long et al., 2016; Sánchez et al., 2021).

Our analyses of behavior during the learning phase focused on checking our assumptions that the participants’ SAIs tracked the target SAI for each condition. That is, we assumed the mean SAI across all learning strides would not differ across conditions (learning SAI mean), but the SAI SD across all learning strides would (learning SAI SD). The purpose of the learning phase was to provide the necessary task practice to develop potential use-dependent biases. The magnitude of use-dependent biases could not be directly measured during learning since other processes were active during this period, cognitive strategies in the case of the Strategy plus Use-Dependent model and Bayesian estimation of visual target location in the case of the Adaptive Bayesian model. Thus, as expected, our models do not make qualitatively different predictions regarding behavior during the learning phase.

Our hypotheses focused on use-dependent biases, probed during the no-feedback washout phase. Use-dependent biases were analyzed at two different time points. First, to characterize the total magnitude of use-dependent learning, we calculated the mean SAI during the first five strides of the washout phase (initial bias). Second, to characterize early changes in use-dependent biases during the washout phase, we calculated the mean SAI of strides 6–30 of the washout phase (early washout; Day et al., 2018; Leech et al., 2018). We also analyzed the rate of washout by regressing subsequent strides onto current strides for the first 50 strides of the washout phase. We report 1-β (slope) as it quantifies the amount of unlearning per stride during the washout phase (Kitago et al., 2013; Wood et al., 2020).

### Model-based methods

We adapted two computational models of use-dependent learning that can explain behavior following training with consistent targets (see below, Simulations); however, the two models make dissociable predictions regarding the effect that changes in movement consistency during learning have on use-dependent biases. We refer to the first model as the Strategy plus Use-Dependent model (S + U). This model was inspired by a previously developed dual-process model of error-based and use-dependent learning (Diedrichsen et al., 2010). Unlike the force-field adaptation task used in the Diedrichsen and colleagues study, the learning paradigm in this study involves, in addition to use-dependent learning, explicit strategies, without contributions from sensorimotor adaptation (French et al., 2018; Wood et al., 2020). Therefore, we replaced the implicit adaptation process from the Diedrichsen model with a strategic process which learns quickly. The second model is referred to as the Adaptive Bayesian model (AB) and was adopted from a reaching study of use-dependent learning (Verstynen and Sabes, 2011).

#### Strategy plus Use-Dependent model

The Strategy plus Use-Dependent model conceptualizes overall motor output as the sum of two parallel processes: cognitive strategy and use-dependent learning. This model attempts to capture the previously reported phenomenon that participants are able to explicitly control SAI in response to VF, yet still demonstrate aftereffects (Long et al., 2016; French et al., 2018; Wood et al., 2020). Strategic learning accounts for the voluntarily controlled component of SAI, while use-dependent learning is insensitive to explicit task goals, and is instead an obligatory stride-by-stride biasing of motor output based purely on recent actions (Diedrichsen et al., 2010). In the context of the current study, the motor output is SAI ( x): the sum of the strategic process ( s) and the use-dependent process ( w) on each stride, n:

(2) $$x_{n+1}=s_{n+1}+w_{n+1}$$

The strategic process corrects errors ( e) between the motor output ( x) and the target ( t):

(3) $$e_{n}=t_{n}-x_{n}$$

(4) $$s_{n+1}=\{\begin{array}{c} A*s_{n}+C*e_{n},with\;VF\\ 0,without\;VF \end{array}$$

0<A<1

0<C<1

This model assumes that individuals remember some proportion, A, of their explicit strategy. For example, when a participant aims for the target, they will remember, to some degree, where they aimed previously. Participants also correct a proportion of the error, C, on each stride. As this is a strategic, or voluntary, process, we assume that s is equal to zero when the VF is turned off and the participants are instructed to walk normally.

Use-dependent learning ( w) occurs in parallel with strategy and becomes biased toward the current motor output ( x). E represents the retention factor for use-dependent learning and F is the use-dependent learning rate. Here, the update is a function of the motor output which, in this experiment, changes based on the error signal, because of strategic learning (Eq. 3), and the slowly evolving use-dependent bias.

(5) $$w_{n+1}=E*w_{n}+F*x_{n}$$

0<E<1

0<F<C

Strategic learning in humans is highly flexible and, under certain conditions, quite rapid (>0.7 in Taylor and Ivry, 2011; Bond and Taylor, 2015). Yet the use-dependent process learns slowly (average learning rate of 0.038 in Diedrichsen et al., 2010). Therefore, we add the constraint that the strategic learning rate, C, must be at least five times faster than the use-dependent learning rate, F. This model also assumes that this learning rate F is fixed and thus, is not sensitive to the consistency of motor output (Diedrichsen et al., 2010). During washout, when the VF is off and there is no strategy, motor output reflects the sole activity of use-dependent learning.

#### Adaptive Bayesian model

In the Adaptive Bayesian model, predicted step length is the weighted combination of expected target locations based on prior experience and current sensory estimates of target location.

Formally, this model follows from Bayes’ theorem and combines the prior expectation of the SAI target ( $\bar{\theta }$) with the current sensory estimate of target position ( θ) to compute the posterior probability distribution. The model assumes that the motor output is a direct readout of the maximum a posteriori (MAP) estimate ( $\theta_{MAP}$) of target location, as in Verstynen and Sabes (2011):

(6) $$\theta_{MAP}=\frac{\sigma_{likelihood}^{2}}{\sigma_{prior}^{2}+\sigma_{likelihood}^{2}}*\bar{\theta }+\frac{\sigma_{prior}^{2}}{\sigma_{prior}^{2}+\sigma_{likelihood}^{2}}*\theta$$

We assume the prior and likelihood are normally distributed. Therefore:

(7) $$\sigma_{posterior}^{2}=\frac{\sigma_{likelihood}^{2}*\sigma_{prior}^{2}}{\sigma_{likelihood}^{2}+\sigma_{prior}^{2}}$$

The mean of the likelihood is centered on the true target location, θ, on each stride, n. The likelihood’s variance, $\sigma_{likelihood}^{2}$, is a free parameter representing the amount of sensory uncertainty regarding target location. During the baseline and washout phases, the target is the participant’s baseline walking asymmetry (0% SAI). We assume that the amount of uncertainty surrounding the participant’s baseline walking is similar to the uncertainty surrounding the visual targets. Therefore, we set the likelihood variance to be consistent throughout the experiment.

As beliefs about the consistency of targets during the learning phase are likely to adjust as more evidence about target locations arrives, use-dependent learning has been more accurately modeled using adaptive priors as compared with a normative Bayesian model that does not include learning of priors (Verstynen and Sabes, 2011). Here, we also assume that the prior will change on a stride-by-stride basis. The adaptive nature of the model is captured by the stride-by-stride updating of the prior probability’s parameters $N({\bar{\theta }}_{n},\sigma_{prior,n}^{2})$:

(8) $${\bar{\theta }}_{n+1}=\left(1-\beta \right)*{\bar{\theta }}_{n}+\beta *\theta_{n}$$

(9) $$\sigma_{prior,n+1}^{2}=\left(1-\beta \right)*\sigma_{prior,n}^{2}+\beta *{\left({\bar{\theta }}_{n}-\theta_{n}\right)}^{2}$$

0<β<1

$$0<\sigma_{likelihood}^{2}<100$$

β is a free parameter representing the learning rate. The Adaptive Bayesian model has two free parameters, in comparison to the four free parameters of the Strategy plus Use-Dependent model.

Our two models provide distinct interpretations of how use-dependent biases evolve and the specific constraints acting on them. The Strategy plus Use-Dependent model assumes separate, yet parallel, explicit (strategy) and implicit (use-dependent) learning mechanisms. In this model, use-dependent learning is persistently active, but evolves slowly in response to the direction of the walking asymmetry. In direct contrast, the Adaptive Bayesian model does not invoke separate explicit and implicit learning processes, but frames the problem of changing an agent’s behavior in response to visual targets (or the absence of them, as during washout) as one of Bayesian estimation (Ernst and Banks, 2002; Körding, 2007; Wei and Körding, 2009; Verstynen and Sabes, 2011). The MAP estimate may certainly result from contributions of implicit and explicit mechanisms, but the model does not distinguish between the two. In this study, the primary comparisons were between the two models’ differing predictions regarding use-dependent biases in response to varying degrees of practice consistency and the empirically observed biases. The Strategy plus Use-Dependent model predicts that the use-dependent bias will be similar across the three different conditions while the Adaptive Bayesian model predicts progressively smaller use-dependent biases as target consistency is reduced.

### Statistical analysis

Model fitting and model selection, in conjunction with behavioral analyses, formed the basis for our inferences regarding which of the two models (hypotheses) was more strongly supported.

#### Computational models

Our competing hypotheses were encapsulated by our two computational models, the Strategy plus Use-Dependent model (model 1) and the Adaptive Bayesian model (model 2), and their corresponding predictions regarding use-dependent biases: the Strategy plus Use-Dependent model predicts no difference in use-dependent bias across conditions, while the Adaptive Bayesian model predicts reduced use-dependent bias during less consistent conditions. We fit both models to individual participant data from all three conditions combined, using the *fmincon* function in MATLAB. This allowed us to obtain one set of parameter values for each individual participant and model. We provide a figure containing individual and group fits for each model and comparisons of simulated biases (using best-fit model parameters) with the behavioral data to further bolster support for one model over the other.

Additional objective support for one model over the other was formally assessed using model selection criteria, specifically Akaike Information Criterion (AIC) scores. We compared these AIC values between the two models using a paired *t* test. Quality of model fits were assessed using R-squared values. The number of subjects best fit by each model are reported and presented in the results section. As fits to individual data can be noisy (Wilson and Collins, 2019), we also calculated AIC scores on fits to the average learning functions across conditions. To provide confidence intervals on parameter estimates, we fit the average learning function for each of 10,000 bootstrapped samples and report the empirical 2.5th and 97.5th percentile values.

#### Behavior

As stated above, we did not have competing hypotheses regarding the learning phase, and we expected participants to accurately follow the visual targets. If this assumption was correct, it would result in learning SAI mean values that would not differ across conditions, but larger learning SAI SD values when going from constant to low variability and high variability conditions. These assumptions were assessed using repeated measures ANOVA and in the case of a significant test, we performed *post hoc* Bonferroni-corrected pairwise comparisons.

As the Adaptive Bayesian model predicts differences in use-dependent bias across conditions, we performed statistical analyses of initial bias, early washout and washout rate using separate repeated measures ANOVAs. In cases of a significant ANOVA, we performed *post hoc* pairwise comparisons with Bonferroni-corrected *t* tests. Because the Strategy plus Use-Dependent model predicts similar use-dependent biases across conditions, we also performed equivalence tests on initial bias, early washout and washout rate using the two one-sided tests (TOST) procedure (Lakens, 2017). Briefly, the TOST procedure involves two composite null hypotheses that an observed effect is either below or above chosen equivalence bounds (Cohen’s *d* of ±0.3; see Lakens, 2013), and thus provides a rigorous means of inferring the lack of a meaningful effect.

We report *t* and *F* statistics, exact *p* values, means, 95% confidence intervals (CIs) and standardized effect sizes (Cohen’s *d* for *t* tests and η_p_^2^ for ANOVAs). For equivalence testing, we also report the empirical equivalence bounds for which we would be able to reject the null hypothesis that there is an effect of condition. Bonferroni corrected *p* values were used for tests involving multiple comparisons. Assumptions of normality and equality of variances were tested with the Shapiro–Wilk test and Levene’s tests, respectively. While all data met assumptions of normality and equality of variances, in case these assumptions were not met, our intention was to perform non-parametric permutation tests. For pairwise comparisons, we intended to use the difference between group means as our test statistic, to be compared with a null distribution created by random shuffling of group assignment in 10,000 Monte Carlo simulations (resampling with replacement), to obtain an exact *p* value. For comparisons involving more than two conditions, we intended to implement a similar approach but use the *F* value obtained from a repeated-measure ANOVA as our test statistic.

In addition to our parametric analyses of preselected epochs, we also employed a cluster permutation analysis to assess SAI differences across the entire washout phase for each condition (Holmes et al., 1996; Maris and Oostenveld, 2007). In this analysis, we compared SAI differences between two conditions at a time with paired *t* tests between bins of three strides. Binning, in this case, was used to mitigate the effects of stride-to-stride SAI variability on the analysis and thereby reduce the probability of a Type II error. The largest cluster of consecutive significant paired *t* tests (*p* < 0.05) was determined and the *t* statistics for this cluster were summed. The summed *t* statistics were then compared with a null distribution of summed *t* statistics. The null distribution is built from resampling each group without replacement 10,000 times and computing the largest cluster’s *t* statistic for each sample. This null distribution served as the null hypothesis which states that each group is sampled from the same distribution. The cluster size from the empirical data were then compared with the null distribution of 10,000 samples. This comparison provided a probability that the empirical cluster was different from the null distribution while controlling for Type I error (Nichols and Holmes, 2002; Maris and Oostenveld, 2007). This analysis was performed three times to compare differences between each condition.

#### Power analysis

We performed an a priori power analysis to determine the sample size required to detect differences in use-dependent biases across conditions, with α of 0.05 and power of 0.90. We estimated a standardized effect size (Cohen’s *d*) of 0.91 using group step asymmetry differences during the early washout phase of experiment 2 from Wood et al. (2020). Based on this estimated effect size, we required 15 participants. However, to ensure we safely exceeded this threshold for power, we recruited 18 participants. This sample size also ensured appropriate counterbalancing of practice schedules across participants while also being well-above the threshold of statistical power documented in comparable motor learning studies (Diedrichsen et al., 2010; Verstynen and Sabes, 2011; Long et al., 2016; French et al., 2018; Wood et al., 2020).

#### Data replacement

Before data collection, we established the following conditions under which we would replace data:

(1) If a participant did not complete the entire learning task for all three conditions because of a technical error or equipment failure in the middle of data collection or if the participant chose to drop out of the experiment.

(2) If the experimenter deemed the participant unsafe to continue the study, which might have occurred because of an injury or illness after the participant was enrolled.

(3) If a participant did not meet a threshold of performance on the task, which was defined as falling outside of 3 SDs from the mean performance in terms of target accuracy. Target accuracy was defined as the mean absolute difference between the target SAI and the actual SAI measured across the entire learning phase.

None of the participants met the criteria for data replacement. However, three participants’ data were removed from our secondary analysis of limb placement because of hip tracking markers falling off or becoming occluded during walking.

#### Model recovery

**Figure 2.**
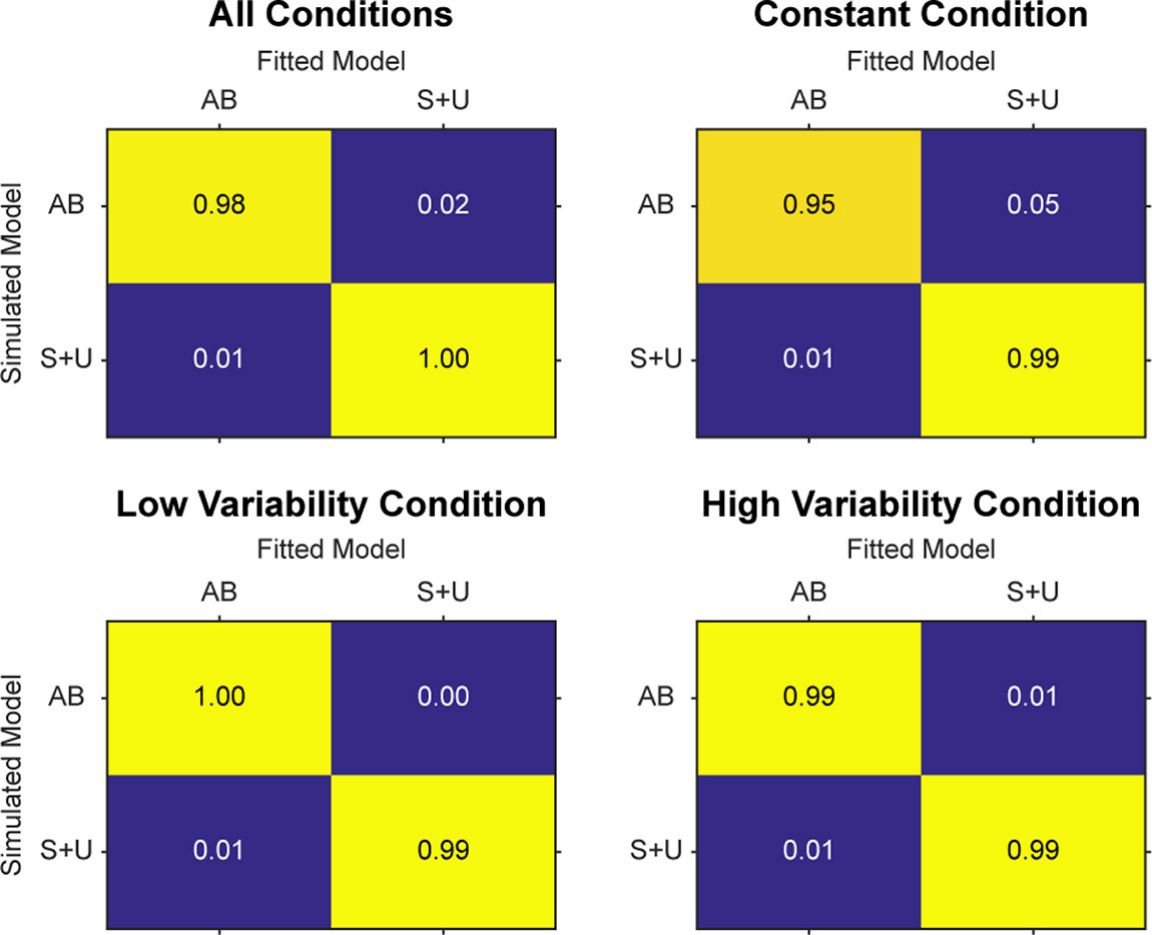
Confusion matrices. Four different confusion matrices for each condition and all conditions combined. Lighter colors indicate higher percentages of better fits for each simulated model. Model fits were compared using AIC. AB is the Adaptive Bayesian model; S + U is the Strategy plus Use-Dependent model.

Because of the central importance of model selection in the proposed study, we performed model recovery analysis on simulated data to (1) confirm that the models are distinguishable under ideal circumstances (Hardwick et al., 2019; Wilson and Collins, 2019) and (2) identify the ideal method of model comparison for this situation [between AIC and Bayesian Information Criterion (BIC); Wilson and Collins, 2019]. We first sequentially simulated data 1000 times per condition with both models using randomized parameter values. We then fit the simulated data with each model, calculating AIC scores for each model fit and directly compared the two values. A confusion matrix summarizes this process, providing the probability that the model which generated the simulated data were better fit by itself or the other model. Ideally, the model that generated simulated data would be better fit by itself than by the other model, resulting in values closer to 1 when comparing the simulations and fits from the same models (Fig. 2, lighter colors on main diagonals) and values closer to 0 when comparing simulations and fits from opposing models (Fig. 2, duller colors on off-diagonals). In Figure 2, we show one confusion matrix for each condition and a combined confusion matrix which reveals that the models are distinguishable under these ideal circumstances when using AIC as the objective model comparison criteria. We performed the same procedure for BIC, however this analysis revealed reduced model discriminability (i.e., smaller range between on-diagonal and off-diagonal values in the confusion matrix). Therefore, this analysis demonstrates that the two models are distinguishable under these constraints and that AIC is better-matched for the current experiment.

#### Simulations

We simulated both models before all data collections to demonstrate how each accounted for the consistency of practiced target step lengths. The Strategy plus Use-Dependent model is robust to environmental consistency in cases, as here, where there is a large asymmetry in one direction. The model assumes use-dependent learning is slower to learn and washout than cognitive strategies; therefore, as long as the practiced asymmetry is much larger than the current state of use-dependent learning, the consistency of target step lengths has minimal impact on its output. The Adaptive Bayesian model stands in direct contrast to this framework. In this model, the MAP estimate, and thus the observed use-dependent bias during washout, is sensitive to environmental consistency: the more consistent (i.e., less variable) the schedule of target step lengths, the more biased toward the prior (i.e., away from the likelihood) the MAP becomes; conversely, the more variable the schedule, the less weight is given to the prior and the more the MAP is pulled toward the likelihood (i.e., the actual target location).

**Figure 3.**
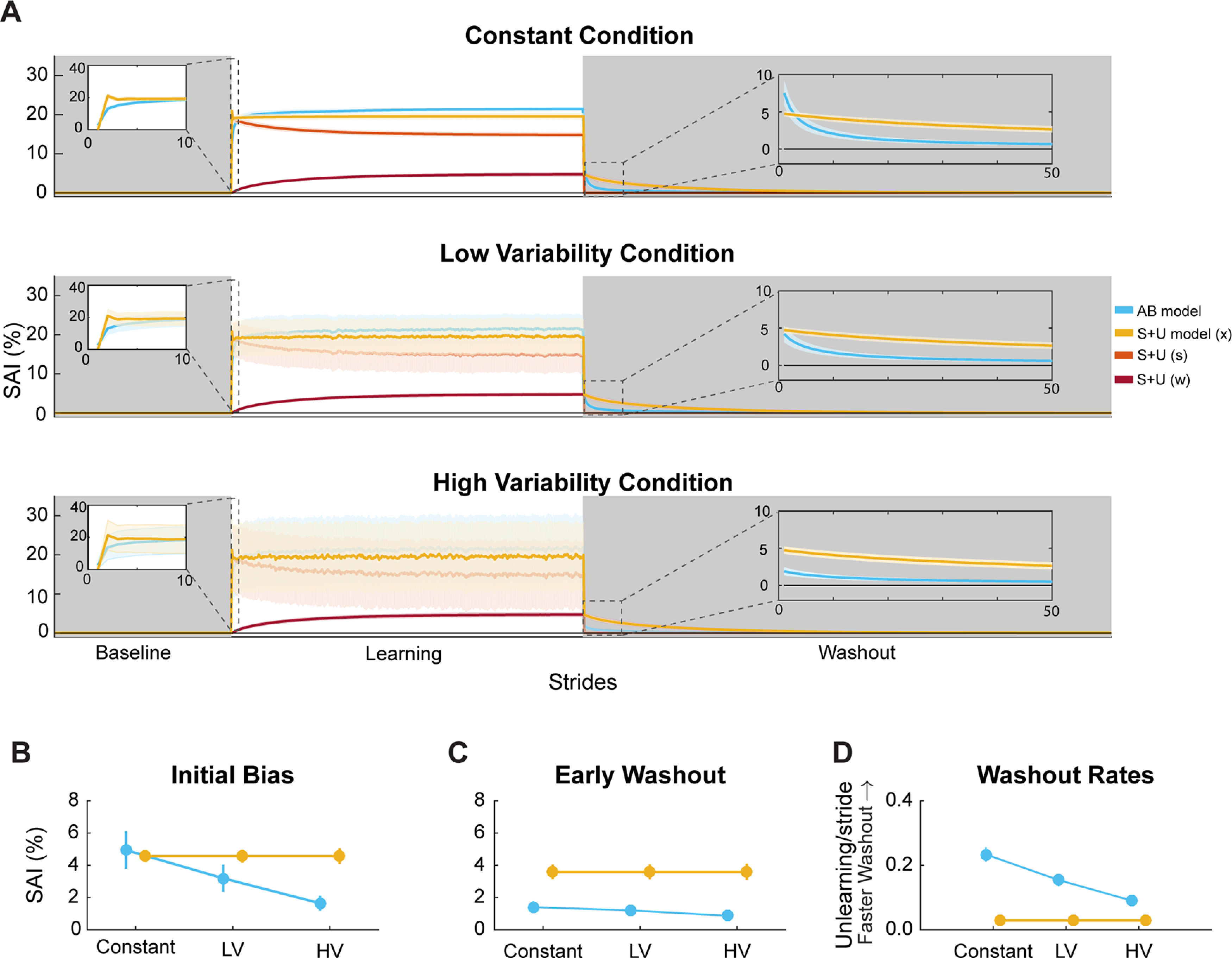
Simulated results. ***A***, Before data collection, the experiment was simulated 1000 times using bootstrapped samples of parameter values from a previously collected dataset (Wood et al., 2020). Results of the simulation are plotted as means with shaded errors indicating SD of bootstrapped sample means. The first 10 strides of the learning phase and the first 50 strides of the washout phase are plotted in the insets. ***B***, Initial bias is the mean of the first five strides of the washout phase. ***C***, Early washout is strides 6–30 of the washout phase. ***D***, Mean and SDs of washout rates for each model across conditions. For panels ***B–D***, filled circles represent the mean, and error bars represent 1 SD of bootstrapped sample means. Some error bars are not visible as their values are small and thus obscured by dots representing mean values. AB model = Adaptive Bayesian model, S + U model = Strategy plus Use-Dependent model, LV = low variability condition, HV = high variability condition.

Preliminary model parameters were obtained by fitting the models to walking data (*n* = 16 participants) from experiment 2 of Wood et al., 2020; which used a protocol most similar to the constant condition in the current study (*R*^2^ values: Adaptive Bayesian model = 0.895 ± 0.019; Strategy plus Use-Dependent = 0.870 ± 0.021; mean ± SEM). We then simulated our proposed experiment 1000 times with the mean learning function from each bootstrapped sample of the individual parameter fits. Figure 3 details the simulated data from these parameters for each condition. The panels in Figure 3*A* show each model simulation for the entire experiment. Across all three conditions, the models diverge in their predictions regarding use-dependent biases during the washout phase.

We plotted the simulated use-dependent biases during both initial bias and early washout (Fig. 3*B*,*C*). Overall, the Strategy plus Use-Dependent model predicts more consistent use-dependent biases across conditions for both initial bias and early washout. However, the Adaptive Bayesian model predicts consistently decreasing bias when the conditions become more variable during the learning phase. For our third point of direct comparison between model predictions, we also analyzed the washout rates for each model (Fig. 3*D*). The Strategy plus Use-Dependent model predicts a consistent washout rate across conditions, whereas the Adaptive Bayesian model predicts slower washout as the conditions during learning increase in variability. Based on these simulations, if the Strategy plus Use-Dependent model is a more accurate model, we should have observed similar use-dependent biases between conditions; however, if the Adaptive Bayesian model is more accurate, we should have observed different use-dependent biases between conditions.

### Changes to Registered Report

We would like to report a few minor changes to the analysis pipeline. First, we originally stated participants would complete the sessions 5–10 d apart; however, because of scheduling constraints four participants completed sessions 13–19 d apart. Second, we originally stated that the first step in the analysis pipeline, filling gaps in the markers, would be performed in Nexus software. However, to properly integrate marker data and visual step length target locations, we exported data directly from our live feedback software, The Motion Monitor, and analyzed all data in MATLAB. To fill gaps, we used MATLAB’s spline function. Third, our original plan was to fit each model to group averaged data and derive bootstrapped 95% CIs from these averaged learning functions. However, averaging across individuals dramatically reduces the differences in variability across the three conditions during the learning phase, thus we only fit the models to individual data. For confidence intervals, we instead bootstrapped individual parameter values to obtain 95% CIs.

## Results

Our aim was to determine how the consistency of movement patterns impacts use-dependent locomotor learning, and our hypotheses were formalized with two computational models that made different predictions about the relationship between the amount of movement variability and the magnitude of use-dependent biases (Fig. 3). We tested these predictions behaviorally by varying step length targets during the learning phase and measuring the amount of use-dependent bias during the washout phase. We will first discuss the behavioral results and then proceed to the model-based analyses.

### Behavioral results

**Figure 4.**
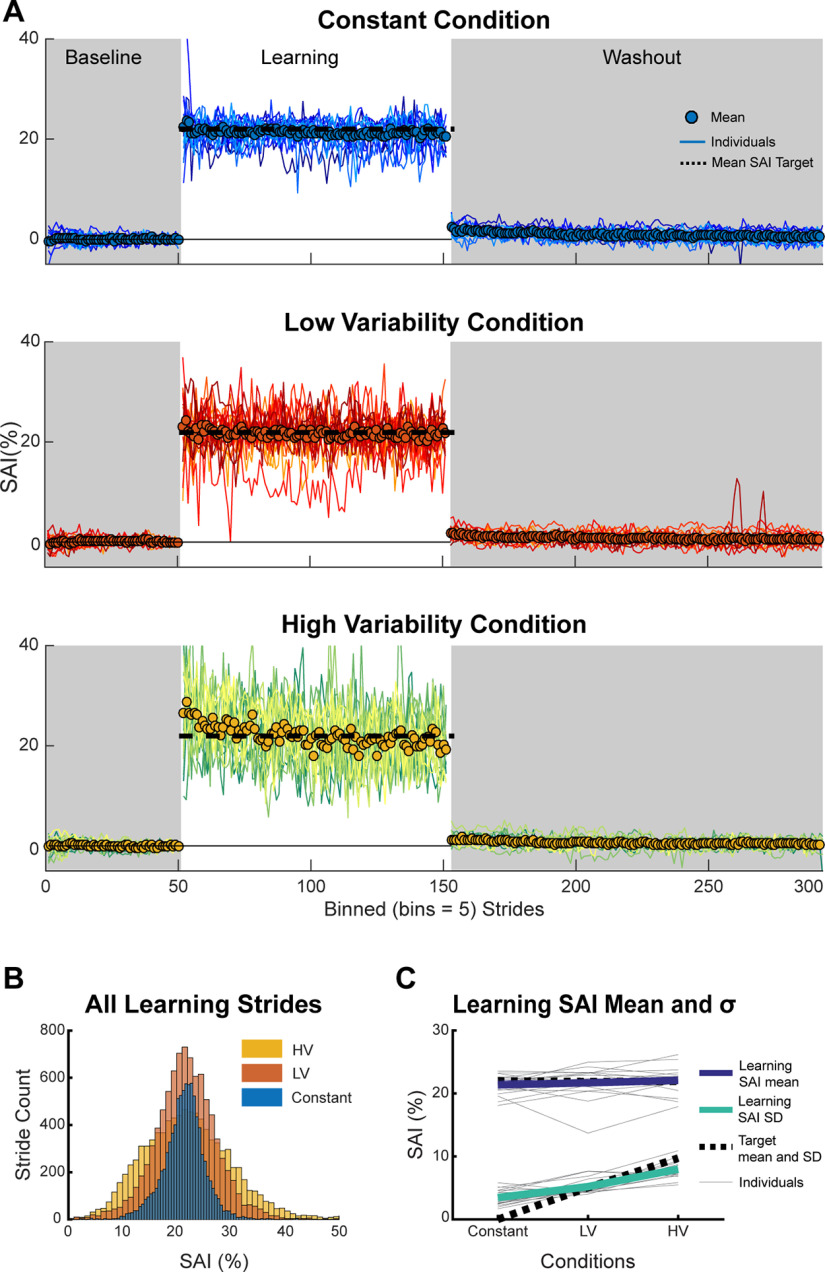
SAI. ***A***, Mean and individual SAI data for all participants in each condition. For plotting purposes, we truncated each phase (baseline, learning, and washout) to match the participant with the fewest strides and binned the data by five strides. ***B***, Histograms of all participant’s SAI values during the learning phase separated by condition. ***C***, Mean (thick lines) and individual (gray lines) learning SAI mean (purple) and learning SAI SD (green) values. The mean and SD of the SAI targets during the learning phase are plotted as black dashed lines for reference. LV = low variability condition, HV = high variability condition.

Eighteen participants (10 female, mean age ± SD = 23.2 ± 3.6 years) completed the constant, low variability, and high variability conditions during different sessions separated by at least 5 d. Using Shapiro–Wilk test and Levene’s tests, we confirmed that all data met assumptions of normality and equality of variances, respectively. Figure 4*A* shows individual and group averaged SAI data for each condition. As expected, the step length targets during the learning phase prompted marked changes in SAI relative to participants’ baseline SAI. These changes occurred rapidly when the VF was introduced highlighting the explicit nature of the task (French et al., 2018; Wood et al., 2020).

**Figure 5.**
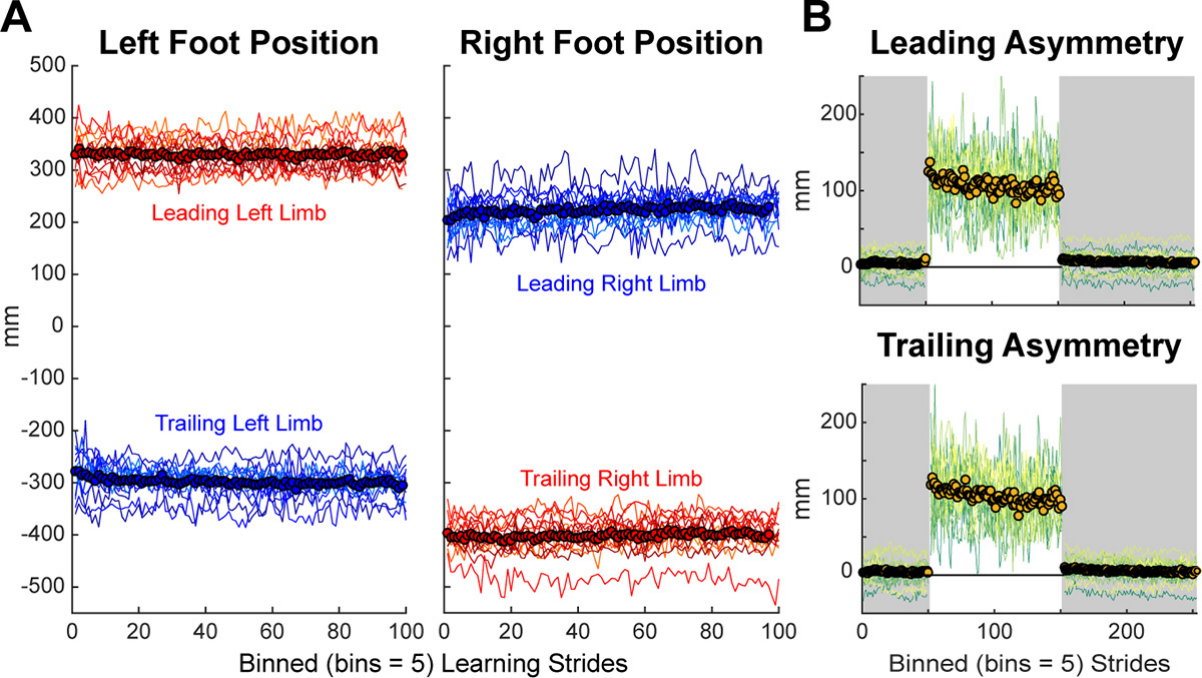
Foot position. ***A***, Mean (circles) and individual (lines) foot position relative to the hip (hip position is 0 on the *y*-axis) for the learning phase of the high variability condition. To hit the (longer) left step length target, participants lengthened both the leading position of their left limb and the trailing position of their right limb relative to the hip (red). The opposite is true for the (shorter) right step length target, where participants shortened both the leading position of the right limb and trailing position of the left limb relative to the hip (blue). ***B***, Mean and individual leading (top panel) and trailing foot placement symmetry (bottom panel) for the high variability condition illustrate the fast nature of limb placement changes. For all panels, we truncated each phase to match the participant with the fewest strides and binned the data by five strides.

To illustrate how individuals made changes in SAI during learning, we calculated leading and trailing limb position. We removed three participants from this analysis because of equipment failure (marker fell off or became covered during the learning phase). We found that participants were able to quickly change their leading and trailing foot positions during the learning phase (Fig. 5*A*) which resulted in an asymmetry in both the leading and trailing limb placement (Fig. 5*B*).

We first assessed whether our experimental manipulation was successful in eliciting similar learning SAI mean values but different learning SAI SD values across all three conditions. This analysis was important to ensure that any potential differences in use-dependent biases were strictly because of movement variability and not because of different magnitudes of learning SAI. It is clear from Figure 4*B*,*C* that participants readily changed their step lengths during the learning phase to closely match the mean of the SAI target distributions, which was always centered around 22% SAI, and we observed no differences across conditions (*F*_(2,17)_ = 1.07, *p* = 0.35, η_p_^2^ = 0.06; mean [95% CI] constant = 21.36 [20.60, 22.13], LV = 21.69 [20.45, 22.94], HV = 22.09 [21.06, 23.13]; Fig. 4*C*, purple). In addition, learning SAI SD increased across each condition in the expected manner (*F*_(2,17)_ = 64.69, *p* = 2.6e^−12^, η_p_^2^ = 0.79; constant = 3.48 [2.86, 4.10], LV = 5.21 [4.64, 5.78], HV = 7.98 [7.31, 8.66]; Fig. 4*C*, green). Specifically, learning SAI SD was greater in the low variability condition compared with the constant condition (*t*_(17)_ = −4.99 *p*_bonf_ = 3.4e^−4^, Cohen’s *d*_z_ = −1.18), greater in the high variability condition compared with the low variability condition (*t*_(17)_ = −7.10 *p*_bonf_ =5.3e^−6^, Cohen’s *d*_z_ = −1.67) and greater in the high variability condition compared with the constant condition (*t*_(17)_ = −9.93 *p*_bonf_ = 5.2e^−8^, Cohen’s *d*_z_ = −2.34). These results indicate that any observed differences in use-dependent biases during the washout phases are because of the effects of movement variability, as opposed to differences in average SAI, during the learning phase.

**Figure 6.**
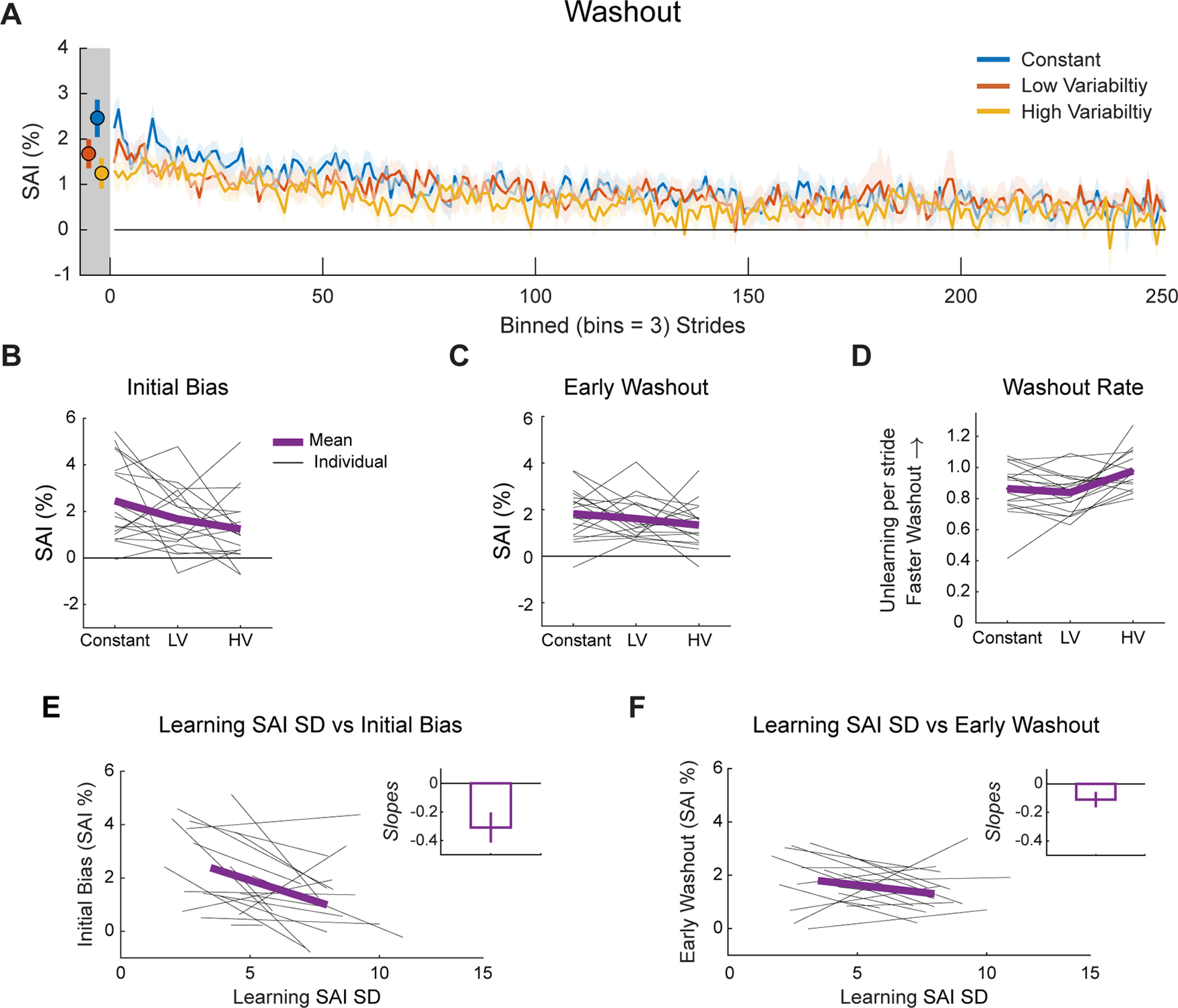
Washout data. ***A***, Mean SAI data during the washout phase of each condition. For plotting purposes, we truncated each individual’s washout phase to match the participant with the fewest strides and binned the data by three strides. The dots and error bars in the gray shaded region represent the mean and SEM of the first five strides of washout for each condition (not binned) which is the same as the initial bias measure. Shaded error bars represent SEM. ***B***, Mean (purple) and individual (gray) initial bias (mean SAI of strides 1–5 during washout) across conditions. ***C***, Mean and individual early washout (mean SAI of strides 6–30 during washout) across conditions. ***D***, Mean and individual washout rate across conditions. ***E***, Exploratory analysis: individual regressions of learning SAI SD and initial bias (gray lines). ***F***, Exploratory analysis: individual regressions of learning SAI SD and early washout (gray lines). In ***E***, ***F***, we used the mean of the individual slopes to calculate the mean regression line (purple). The mean slopes are plotted in the insets. Error bars represent SEM. LV = low variability condition, HV = high variability condition.

Our primary comparisons centered on use-dependent biases during the washout phase, when participants were asked to walk normally and no VF was provided on the screen. As a whole, participants were not immediately able to return to walking normally during the washout phase. Instead, they demonstrated strong evidence of use-dependent learning in the form of a bias in the direction of the repeated movements made during the learning phase (Fig. 6*A*). We compared initial bias across conditions to determine whether movement variability during learning constrained the total magnitude of use-dependent learning. We found that, overall, initial biases decreased as movement variability increased (*F*_(2,17)_ = 4.23, *p* = 0.02, η_p_^2^ = 0.20; Fig. 6*B*), with reliable differences in initial bias between the constant and high variability conditions (*t*_(17)_ = 2.78, *p*_bonf_ = 0.04, Cohen’s *d*_z_ = 0.65). The other pairwise contrasts between constant and low variability (*t*_(17)_ = 1.83, *p*_bonf_ = 0.25, Cohen’s *d*_z_ = 0.43) conditions and low and high variability conditions (*t*_(17)_ = 1.06 *p*_bonf_ = 0.91, Cohen’s *d*_z_ = 0.25) were not reliable. These results demonstrate that the total magnitude of use-dependent learning measured in the first five strides of normal walking, when going from constant to high variability conditions, depends on the amount of movement variability.

While the magnitude of use-dependent learning, measured as initial biases, depended on movement variability, this dependency quickly diminished during washout. As visible in Figure 6*C*, early washout did not reliably depend on condition (*F*_(2,17)_ = 1.26, *p* = 0.30, η_p_^2^ = 0.07). We additionally performed cluster permutation analyses to determine whether there were differences between conditions across the entire washout phase. However, none of the three comparisons resulted in cluster sizes greater than three. As there were no significant differences between the conditions, we ran equivalence tests between each condition using the TOST procedure with equivalence bounds set at a Cohen’s *d*_z_ = ± 0.3. Although the first two analyses of early washout suggest that there were no differences, we also found no evidence of equivalence between the three conditions using the TOST procedure (all *t* scores ≤ 0.19, all *p*s ≥ 0.29). Combined, these results suggest that despite an initial reliance on movement variability, differences in use-dependent biases were not sustained throughout the washout phase.

Using an autoregression analysis, we found differences in washout rate across the conditions (*F*_(2,17)_ = 7.21, *p* = 2.5e^−3^, η_p_^2^ = 0.30; Fig. 6*D*), with a faster decay occurring in the high variability condition compared with the low variability condition (*t*_(17)_ = −3.46, *p*_bonf_ = 0.01, Cohen’s *d*_z_ = −0.82). No differences were found between the washout rates of the constant and low variability (*t*_(17)_ = 0.86, *p*_bonf_ = 1.21, Cohen’s *d*_z_ = 0.20) or constant and high variability conditions (*t*_(17)_ = −2.47, *p*_bonf_ = 0.07, Cohen’s *d*_z_ = −0.58). This idiosyncratic pattern of washout rates (high variability > constant > low variability) is at odds with the Strategy plus Use-Dependent model prediction of constant washout rates and the Adaptive Bayesian model prediction of washout rates that diminish with increased movement variability (Fig. 2*D*). However, as the amount of stride-by-stride variability was, for many participants, greater than their mean bias during the analysis window, the estimated washout rates were highly sensitive to noise. This led to overestimation of unlearning rates across all three conditions.

#### Exploratory analysis

##### Relationship between use-dependent learning and movement variability

Although, at the group level, learning SAI SD increased in the predicted manner based on condition, this relationship did not hold at the individual level for every participant. In other words, not every participant’s learning SAI SD increased in an orderly manner from constant through high variability conditions. Therefore, we completed a more granular analysis of how movement variability impacted use-dependent learning by regressing each individuals’ initial bias against their learning SAI SD, yielding a slope coefficient for each individual (Fig. 6*E*). We compared these slope coefficients to 0 using a *t* test and found a consistent inverse relationship between learning SAI SD and initial bias (mean slope = −0.31 [−0.53, −0.09], *t*_(17)_ = −2.94, *p* = 0.01), indicating that the more variable a participant’s SAI was across learning, the smaller their total magnitude of use-dependent learning. A similar trend was also observed between learning SAI SD and early washout (mean slope = −0.11 [−0.22, 0.01], *t*_(17)_ = −2.02, *p* = 0.06; Fig. 6*F*), albeit with a sharp reduction in the slope coefficients. These results bolster the group-level analysis: there is a strong relationship between movement variability and use-dependent learning measured by initial bias, but those differences quickly dissolve over subsequent strides.

To further validate the idea that observed changes in use-dependent biases were because of differences in movement variability rather than the magnitude of practiced asymmetry during learning, we applied the same analytical approach to learning SAI mean data. Consistent with this view, we found no relationship between learning SAI mean and initial bias (mean slope = 0.15 [−0.95, 1.24], *t*_(17)_ = 0.28, *p* = 0.78) or between learning SAI mean and early washout (mean slope = −0.45 [−1.29, 0.39], *t*_(17)_ = −1.14, *p* = 0.27). Together, this exploratory analysis offers strong support that movement variability impacts initial biases, but that this effect quickly dissipates.

### Model-based results

**Figure 7.**
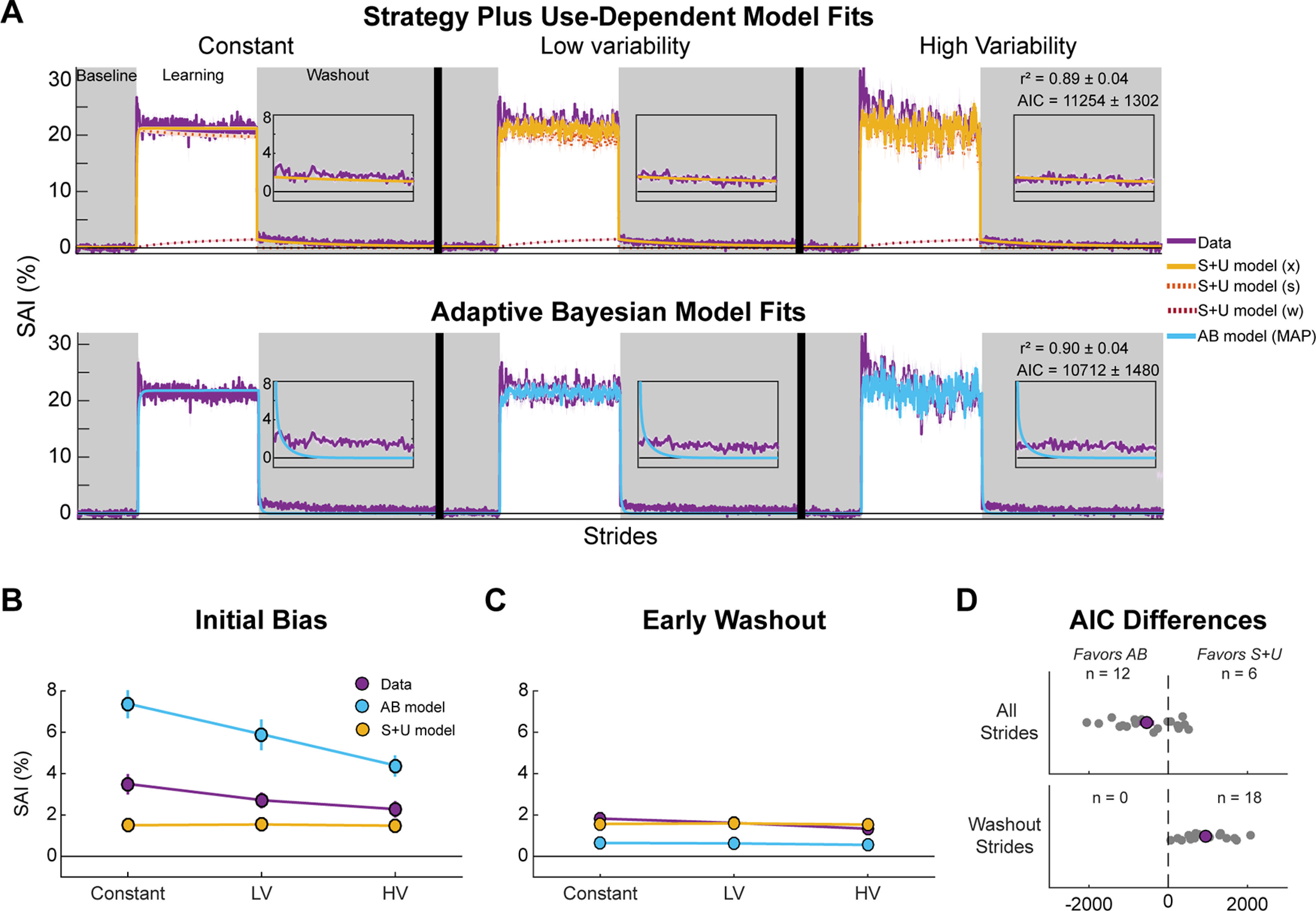
Model-based results. We fit each model to each individual’s SAI data and simulated the models using the best fit parameter values. We plot the mean of the model simulations with the mean of the behavioral data (purple). ***A***, Strategy plus Use-Dependent (S + U model; top row) and Adaptive Bayesian (AB model;bottom row) simulations together with behavioral data. Gray regions indicate no VF phases. Insets show the first 100 strides of the washout phase. ***B***, Mean initial bias for each model and empirical data across conditions. ***C***, Mean early washout for each model and behavioral data across conditions. ***D***, Individual (gray dots) and mean (purple dot) differences between AICs for each model plotted as Adaptive Bayesian model minus Strategy plus Use-Dependent model. Dots to the left of the dotted line indicate better fits to the Adaptive Bayesian model with the number of participants included above. Top shows the AIC differences when considering all strides. Bottom shows the AIC differences when considering only the washout strides (exploratory). Shaded error and error bars represent SEM. Extended Data Figures 7-1, 7-2 show the individual fits for the Strategy plus Use-Dependent and Adaptive Bayesian models, respectively. LV = low variability condition, HV = high variability condition.

Overall, the behavioral findings support the hypothesis that the total magnitude of use-dependent learning depends on movement variability, consistent with the Adaptive Bayesian model predictions (Fig. 3). However, the weak dependence on movement variability during early washout appears better-aligned with the Strategy plus Use-Dependent model. Thus, to objectively assess support for one computational model over the other, we directly compared fits of the two models to the behavioral data. We fit both models to the SAI data of each individual and plotted the mean of these simulations against the group averaged data (Fig. 7; individual fits are included as Extended Data Figs. 7-1, 7-2; Tables 1, 2 show bootstrapped parameter values). While both models demonstrated good fits to individual data (S + U mean ± SD *r*^2^ = 0.89 ± 0.04, AB *r*^2^ = 0.90 ± 0.04), AIC scores indicate stronger support of the Adaptive Bayesian model than the Strategy plus Use-Dependent model (S + U model AIC = 11,254 [10,607, 11,902]; AB model AIC = 10,712 [9976, 11,448], *t*_(17)_ = 2.90, *p* = 9.8e^−3^, Cohen’s *d*_z_ = 0.68; Fig. 7*D*, top).

10.1523/ENEURO.0265-20.2021.f7-1 Extended Data Figure 7-1 Individual Strategy plus Use-Dependent model fits. The order in which each participant completed the conditions is on the *x*-axis. The *r*^2^ and AIC values for each fit are also provided. Download Figure 7-1, TIF file.

10.1523/ENEURO.0265-20.2021.f7-2 Extended Data Figure 7-2 Individual Adaptive Bayesian model fits. The order in which each participant completed the conditions is on the *x*-axis. The *r*^2^ and AIC values for each fit are also provided. Download Figure 7-2, TIF file.

**Table 1:** Parameters for the Adaptive Bayesian models

Model	β (VF)	σ_likelihood_ (VF)	β (no VF)	σ_likelihood_ (no VF)
AB	0.22 [0.17, 0.29]	63.9 [45.86, 78.54]	NA	NA
Modified AB	0.20 [0.15, 0.28]	78.86 [62.78, 89.69]	1.0e^−3^ [4.6e^−4^, 2.0e^−3^]	2.05 [0.74, 4.78]

Bootstrapped means and 95% confidence intervals. VF = visual feedback; NA = not applicable.

**Table 2:** Parameters for the Strategy plus Use-Dependent models

Model	C	A	F	E	γ _LV_	γ _HV_
S + U	0.46 [0.38, 0.55]	0.99 [0.98, 0.99]	5.0e^−4^ [2.8e^−4^, 1.3e^−3^]	0.95 [0.77, 1.0]	NA	NA
Modified S + U	0.46 [0.38, 0.55]	0.99 [0.97, 0.99]	2.9e^−3^ [3.6e^−4^, 1.3e^−2^]	0.94 [0.97, 1.0]	0.76 [0.59, 0.87]	0.53 [0.36, 0.69]

Bootstrapped means and 95% confidence intervals. LV = low variability condition; HV = high variability condition; NA = not applicable.

#### Exploratory analysis. Model fits to use-dependent biases only

While model selection criteria support the Adaptive Bayesian model, we noticed that this model does a poor job of capturing the slow return to baseline SAI (washout insets and Fig. 7*C*). In contrast, the Strategy plus Use-Dependent model, while missing the dependence on movement variability (Fig. 7*B*), more accurately captures the similar biases across conditions during early washout and beyond (washout insets and Fig. 7*C*). As the aim of this study was to determine the effects of movement variability on use-dependent biases observed during washout, we calculated AIC scores for only the washout strides. These washout-only AIC scores supported the Strategy plus Use-Dependent model (washout only AICs: S + U model = 1810 [1486, 2134]; AB model = 2793 [2561, 3026]; *t*_(17)_ = −7.65, *p* = 6.7e^−7^, Cohen’s *d*_z_ = −1.80; Fig. 7*D*, bottom). Based on these discrepancies, we made *post hoc* adjustments to each model to better understand and capture both phenomena.

#### Exploratory analysis. Modified models

The original models predicted a unitary process that either would or would not show sensitivity to movement variability throughout the entire washout. We did not originally account for the presence of what appears to be two distinct features of use-dependent locomotor learning that emerged from the behavioral data: the immediate sensitivity to movement variability seen in the initial biases coupled with the later component of use-dependent biases that were similar in magnitude across conditions and therefore appear insensitive to practice consistency. As such, we modified both models in an attempt to capture these combined effects.

One reason the Strategy plus Use-Dependent model likely did not capture the dependence of initial bias on movement variability is the constant use-dependent learning rate (*F*). Therefore, to capture the empirically observed sensitivity to movement variability, we added a condition-specific gain (γ_Cond_) that acts on the use-dependent learning rate:

(10) $$w_{n+1}=E*w_{n}+\gamma_{Cond}*F*x_{n}$$

Our assumption was that the learning rate diminishes with increased movement variability. Therefore, γ_Cond_ was set to 1 during the constant condition and was constrained to decrease in an ordinal manner across the low and high variability conditions. This gain is analogous to implementations used in other models in which learning rates are adjusted based on prior movement history (Herzfeld et al., 2014; McDougle et al., 2017; Kim et al., 2019).

For the Adaptive Bayesian model, we fit different $\sigma_{likelihood}^{2}$ and prior learning rate, β, parameters for the phase with VF (learning) and those without (baseline and washout). This modified model assumed that the two conditions are distinct, such that sensory uncertainty and learning of prior probabilities regarding where to step is impacted by the presence (or absence) of VF.

**Figure 8.**
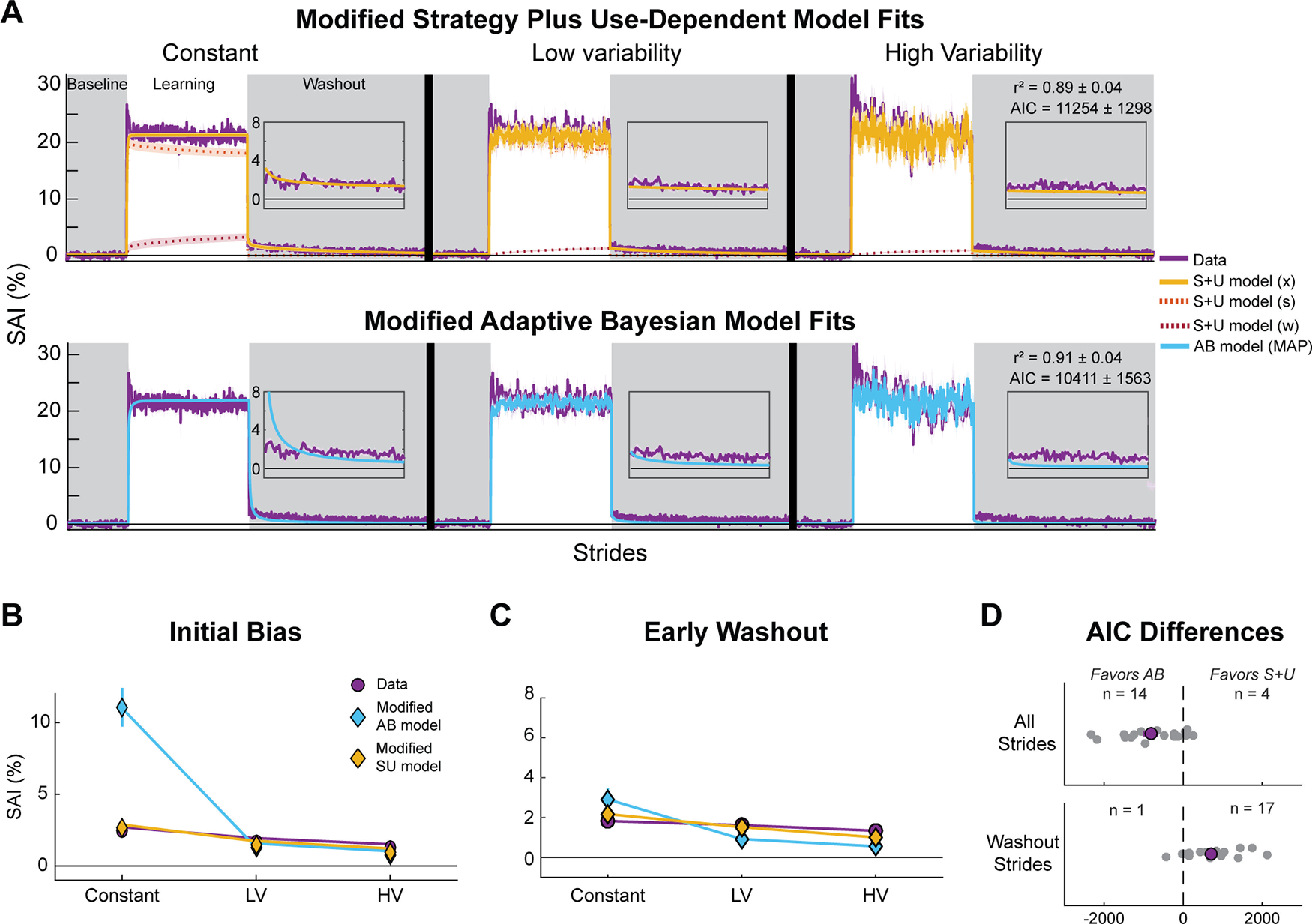
Modified model-based results. We fit each modified model to each individual’s SAI data and simulated the models using the best fit parameter values. We plot the mean of the model simulations with the mean of the behavioral data (purple). ***A***, Modified Strategy plus Use-Dependent (S + U; top row) and modified Adaptive Bayesian (AB; bottom row) simulations together with behavioral data. Gray regions indicate no VF phases. Insets show the first 100 strides of the washout phase. ***B***, Mean initial bias for each model and empirical data across conditions. ***C***, Mean early washout for each modified model and behavioral data across conditions. ***D***, Individual (gray dots) and mean (purple dot) differences between AICs for each model plotted as modified Adaptive Bayesian model minus modified Strategy plus Use-Dependent model. Dots to the left of the dotted line indicate better fits to the modified Adaptive Bayesian model with the number of participants included above. Top shows the AIC differences when considering all strides. Bottom shows the AIC differences when considering only the washout strides. Shaded error and error bars represent SEM. Extended Data Figures 8-1, 8-2 show the individual fits for the modified Strategy plus Use-Dependent and modified Adaptive Bayesian models, respectively. LV = low variability condition, HV = high variability condition.

We performed the same fitting and plotting procedures for the adjusted models as we did for the original models (Fig. 8; individual fits are included as Extended Data Figs. 8-1, 8-2; Tables 1, 2 show bootstrapped parameter values). The modified models accounted for a high proportion of observed variance, similar to their original counterparts (modified S + U *r*^2^ = 0.89 ± 0.04; modified AB *r*^2^ = 0.91 ± 0.04). Once again, there was stronger objective support for the modified Adaptive Bayesian model overall as compared with the modified Strategy plus Use-Dependent model (modified S + U model AIC = 11,220 [10,574, 11,865]; modified AB model AIC = 10,411 [9634, 11,189], *t*_(17)_ = 4.34, *p* = 4.4e^−4^, Cohen’s *d*_z_ = 1.02; Fig. *8D*, top). The modifications generally improved the AIC scores, with reliable differences between the two versions of the Strategy plus Use-Dependent models (*t*_(17)_ = 2.35, *p* = 0.03, Cohen’s *d*_z_ = 0.55) and the Adaptive Bayesian models (*t*_(17)_ = 6.82, *p* = 3.0e^−6^, Cohen’s *d*_z_ = 1.61), indicating that the modifications did not result in overfitting the data.

10.1523/ENEURO.0265-20.2021.f8-1 Extended Data Figure 8-1 Individual modified Strategy plus Use-Dependent model fits. The order in which each participant completed the conditions is on the *x*-axis. The *r*^2^ and AIC values for each fit are also provided. Download Figure 8-1, TIF file.

10.1523/ENEURO.0265-20.2021.f8-2 Extended Data Figure 8-2 Individual modified Adaptive Bayesian model fits. The order in which each participant completed the conditions is on the *x*-axis. The *r*^2^ and AIC values for each fit are also provided. Download Figure 8-2, TIF file.

Again, based on AIC scores, the modified Adaptive Bayesian model performed better than the modified Strategy plus Use-Dependent model. This was because of the modified Adaptive Bayesian model’s ability to capture the fluctuations in SAI during the learning phase better than the modified Strategy plus Use-Dependent model. Looking at Figure 8*D*, it is clear that when we focused our analyses on use-dependent biases during the washout phases, AIC scores again favored the modified Strategy plus Use-Dependent model (washout only AICs: modified S + U model = 1638 [1238, 2039]; modified AB model = 2469 [2237, 2701]; *t*_(17)_ = −5.87, *p* = 1.8e^−5^, Cohen’s *d*_z_ = −1.38; Fig. 8*D*, bottom). Furthermore, the insets of Figure 8*A*, top row, clearly show that the modified Adaptive Bayesian model still struggled to capture the slow decay of use-dependent biases during the washout phase. In contrast, the modified Strategy plus Use-Dependent model hewed closely to the behavioral data during washout (washout insets). As a result, the modified Strategy plus Use-Dependent model generated initial biases and early washout predictions that more closely matched the behavioral data than the modified Adaptive Bayesian model (Fig. 8*B*,*C*).

## Discussion

Use-dependent learning has been reported across reaching (Diedrichsen et al., 2010), single-joint thumb (Classen et al., 1998; Mawase et al., 2017), and wrist (Selvanayagam et al., 2016; Marinovic et al., 2017) movements, and saccades (Reuter et al., 2019). However, surprisingly little attention has been paid to use-dependent learning during locomotion, a naturally repetitive, cyclical form of movement. Here, we directly tackled the question of how the consistency of locomotor movement patterns impacts use-dependent learning. We compared two competing computational accounts of use-dependent biases by systematically varying environmental consistency during learning and assessed the state of use-dependent biases during a no-feedback washout block. We found that use-dependent biases were initially dependent on the amount of movement variability during learning, consistent with the Adaptive Bayesian model. However, to our surprise, this dependence faded quickly as biases became similar in magnitude over subsequent strides across all conditions, an observation more consistent with the two-process, Strategy plus Use-Dependent model. Simple *post hoc* adjustments to these models, specifically the Strategy plus Use-Dependent model, made clear that these combined effects result from a unitary use-dependent learning process.

### Alternative explanations

One alternative explanation to the differences in aftereffects, which we labeled as use-dependent biases, is that they are actually because of the reinforcement of successful actions rather than movement repetition (Codol et al., 2018; Holland et al., 2018). We did not provide any explicit reward in our task, but it is possible that an implicit target hitting signal could have induced an implicit aftereffect (Kim et al., 2019; Leow et al., 2020). However, there was no reliable positive relationship between the proportion of target hits and initial bias across participants, indicating the aftereffects were likely because of movement repetition rather than reinforcement of successful actions.

Another alternative explanation is that the reduced aftereffects during the high variability condition are because of lower SAI at the end of the learning phase (Verstynen and Sabes, 2011; Marinovic et al., 2017; Wood et al., 2020), possibly because of systematic sampling bias of SAI targets or fatigue. Indeed, when looking at the group behavior in Figure 4, it does appear that the mean SAI during the high variability condition decreases during the learning phase. However, this apparent decline was because participants’ SAI, on average, overestimated target step lengths and at the beginning of learning and later converged toward the mean target SAI of 22% near the midway point of the learning phase. It is also possible that fatigue played a role, but target hits were similar at the beginning, middle and end of the learning phase. Critically, when examining the averages of both target SAI and observed SAI over the last 50 strides of learning for each participant, we found no reliable differences between conditions. These findings, along with the consistent relationship we observed between learning SAI SD and initial bias indicates that movement variability was the driving force behind the varying magnitudes of initial bias across conditions, not target sampling bias or fatigue.

### Differences in initial biases because of movement variability

We saw that initial SAI biases decreased as the practice during learning became more variable. This observation aligns with previous studies of upper-extremity movements, in which movement biases are largest with consistent target presentations and diminish as targets become more variable (Jax and Rosenbaum, 2007; Verstynen and Sabes, 2011; Marinovic et al., 2017). In the Adaptive Bayesian models (original and modified), this sensitivity to movement variability is represented by the likelihood uncertainty and the learning rate for adapting priors, which in turn affects the prior uncertainty. As movement variability increases, prior uncertainty increases. Therefore, the posterior becomes more biased toward the sensory observation, i.e., the likelihood, which is centered on baseline SAI during washout, resulting in a smaller initial bias.

The original Strategy plus Use-Dependent model, which has a fixed use-dependent learning rate, could not explain the differences in initial biases. However, by adding a gain on the use-dependent learning rate that is sensitive to movement variability, we observed high-quality fits to the initial biases as well as the entire washout phase. Others have suggested that sensitivity to movement variability may be because of the dialing up or down of excitability within specific circuits of the motor cortex because of repeated movements, an instantiation of Hebbian plasticity (Hebb, 1949; Verstynen and Sabes, 2011). Conceptually, the idea of increased sensitivity to movement variability, represented by the gain factor in the modified Strategy plus Use-Dependent model, is similar to other computational models of motor learning in which recent movement history affects the weights applied to different learning units (Herzfeld et al., 2014; McDougle et al., 2017; Kim et al., 2019).

### After initial bias, use-dependent biases were consistent across conditions

Despite an initial sensitivity to movement variability, use-dependent biases quickly became similar in magnitude across conditions over subsequent strides. Why were the Adaptive Bayesian models unable to capture this effect? The shifting of the prior toward baseline SAI as well as the likelihood being centered on baseline SAI during washout leads to an inevitable decay toward zero SAI at a much faster rate than empirically observed. The modified Adaptive Bayesian model showed a slight improvement over the original model, with a slower decay. One might expect this to occur from an increase in sensory uncertainty (σ_likelihood_) when there is no VF of target SAIs, as during baseline and washout. The end result being a greater bias toward the prior distribution, centered near the 22% target SAI at the start of washout. However, the estimated sensory uncertainty was actually much lower when there was no VF compared with when VF was provided during learning (Table 1). This suggests that participants have greater sensory confidence in what their baseline SAI should be in the absence of VF, analogous to our high confidence in proprioceptive judgments of body midline (Clayton et al., 2014). Rather than sensory uncertainty, the slower overall decay was primarily because of a much more slowly updating prior during washout. This led to a slower shift toward the sensory observation centered on baseline SAI. However, even in the modified version of the Adaptive Bayesian model, the dynamics are such that there is a strong pull toward baseline SAI, and over strides, there is no countervailing force to sustain the bias.

Unlike the Adaptive Bayesian models, the modified Strategy plus Use-Dependent model was successful in capturing the way use-dependent biases quickly became similar in magnitude across conditions following the first several strides. Our use of a variability-sensitive gain served to change the effective learning rates (γ_Cond_ * *F*). This in turn resulted in a unitary learning process that could start at different points but quickly reach equal magnitudes given different learning rates. The dynamic balance between learning and forgetting in the use-dependent process, both of which are always active during volitional movement, results in convergence at asymptote. A similar dynamic equilibrium between learning and forgetting has been reported in the reaching literature and used to explain asymptotic learning in response to visuomotor errors (Vaswani et al., 2015; Morehead and Smith, 2017; Kim et al., 2018).

Another core feature of the Strategy plus Use-Dependent models is that, unlike the Adaptive Bayesian models, they show a sustained bias throughout the washout (Figs. 7, 8). This slow, sustained decay may be a behavioral signature of use-dependent learning, distinguishing it from other implicit forms of learning like sensorimotor adaptation, and has been reported before in reaching (Diedrichsen et al., 2010; Bains and Schweighofer, 2015). In the current study, we showed that despite varying levels of movement variability during learning, the use-dependent bias will accumulate over strides. Because the use-dependent learning process in this model learns and forgets slowly, it can effectively filter out the stride-by-stride fluctuations around an average movement direction. With a large overall movement bias in a consistent direction, as occurs during the learning phases of all conditions, the slow use-dependent learning process will stubbornly proceed. And during washout, the use-dependent process resists returning to baseline SAI through the combination of a high retention factor and the continuous learning from the non-zero SAI associated with the current stride.

### Fast strategic adjustments during the learning phase

As our modeling analyses of the washout phases made clear, the Strategy plus Use-Dependent models provide a better explanation of pure use-dependent biases. However, when examining simultaneous model fits to all of the behavioral data, the Adaptive Bayesian models were favored overall by objective model selection criteria (Figs. 7*D*, 8*D*). This could only be because of the Adaptive Bayesian models’ advantage over the Strategy plus Use-Dependent models during learning, specifically, their ability to more accurately capture the rapid fluctuations in SAI during learning.

A key feature of the Adaptive Bayesian models is that they combine the target expectation (prior) with sensory information (likelihood) regarding the current target location, with the likelihood centered around the actual target location. Therefore, the posterior is always biased toward the true target step length and can quickly adjust on a stride-by-stride basis, as we would expect human participants to be able to. Conversely, the Strategy plus Use-Dependent models correct for a proportion of the distance between the target and the actual motor output based on the strategic correction rate (C), which is coupled to a retention factor (A). Given that the mean strategic correction rate in our sample was 0.46, this indicates that the strategic component of the model will not be able to make nearly as rapid changes, and it will also not start off with an immediate bias toward the target step length, as the Adaptive Bayesian models do.

Do these results mean that visual-guided locomotor stepping is better thought of as Bayesian decision-making than voluntary, strategic updating? No, since these processes are not mutually exclusive, especially in the context of motor control (Wolpert and Landy, 2012). A more parsimonious interpretation is that cognitive strategies during learning may be best represented by a Bayesian learner. In other words, the fast movement adjustments required to hit the visual targets during learning may be most accurately conceptualized as an optimal decision-making process of where to step based on current and prior sensory (target) information. However, the question of how Bayesian estimation interacts with a parallel use-dependent learning process should be taken up in a future study.

In conclusion, our two distinct hypotheses, encapsulated by the Strategy plus Use-Dependent and Adaptive Bayesian models, provide us with unique insights into how movement variability constrains locomotor use-dependent learning. The modified Strategy plus Use-Dependent model made clear that variability may impact the learning rate of the use-dependent process, the signature of which is the biasing of action execution based on movement history. This in turn leads to an initial bias that is correlated with practice consistency. The apparent sensitivity to practice consistency will soon fade, however, and what is left is a small but sustained aftereffect that is resistant to completely washing out (Diedrichsen et al., 2010; Bains and Schweighofer, 2015).

Although a secondary finding, both Adaptive Bayesian and Strategy plus Use-Dependent models further support the idea that visually-guided walking requires a highly flexible action selection process (Wood et al., 2020). The Adaptive Bayesian models indicate that this action selection process may be best characterized as Bayesian decision-making. The dichotomy between action selection and action execution has been an active area of investigation within the sensorimotor learning literature of the past decade (Taylor and Ivry, 2012; McDougle et al., 2016; Kim et al., 2021). However, it is only more recently that a similar split has been observed in studies of use-dependent learning, with demonstrations that recent movement history can quickly elicit large action selection biases while action execution biases take longer to develop and are much smaller (Marinovic et al., 2017; Reuter et al., 2019). The current study suggests visually-guided walking requires the parallel operation of a flexible action selection process and a slow, rigid use-dependent learning one.

Our findings have significant implications for the study of locomotor learning, as repeating movement patterns that are different from baseline walking is a general feature of nearly all locomotor learning studies (Roemmich and Bastian, 2015; Day et al., 2018; Sánchez et al., 2021). For example, in split belt treadmill walking adaptation paradigms, perhaps the most well-studied form of locomotor learning (Dietz et al., 1994; Reisman et al., 2005; Torres-Oviedo et al., 2011; Roemmich et al., 2016), participants repeat, at asymptote, an adapted motor plan that necessitates dramatic changes in the temporal and spatial features of their normal gait pattern (Malone et al., 2012; Finley et al., 2015). Despite this ubiquity of repetition during split-belt adaptation, the role of repetition remains murky (Roemmich and Bastian, 2015). Our present study suggests use-dependent learning may play an important, yet unrecognized, role in the overall behavioral changes elicited by split-belt walking. In addition, repetition is a primary component of rehabilitation practices (Kleim and Jones, 2008; Hornby et al., 2019), but to what extent repetition during walking contributes to altered gait patterns, whether adaptive or maladaptive, remains unknown. We hope the present study motivates future work that will unravel these interactions and shed more light on this fundamental learning process.

## Synthesis

Reviewing Editor: Alexxai Kravitz, Washington University in St. Louis Decisions are customarily a result of the Reviewing Editor and the peer reviewers coming together and discussing their recommendations until a consensus is reached. When revisions are invited, a fact-based synthesis statement explaining their decision and outlining what is needed to prepare a revision will be listed below. The following reviewer(s) agreed to reveal their identity: NONE.

I am very sorry for the long delay in returning a decision on this. This was not what I planned on. Your stage 1 manuscript was reviewed last time by two reviewers. I invited them both to review this stage 2 report and one declined while the other accepted. Speaking with editorial staff we decided to go with the one reviewer and myself, and not attempt to get a new one. Unfortunately the other reviewer never returned their review, and has stopped responding to my emails. So I have reviewed your manuscript and the changes you outlined to the original plan, and found both to be excellent. I appreciate you sticking with the registered report format, and I wish I had done this a month ago and gotten this decision back to you! At any rate I am accepting your manuscript as is. Once again, sorry for the delay.
