# Supplementary figures and images for: The Consistency of Prior Movements Shapes Locomotor Use-Dependent Learning
Source: eNeuro. 2021 Sep 8;8(5):ENEURO.0265-20.2021. doi: 10.1523/ENEURO.0265-20.2021 (PMC8431821; doi:10.1523/ENEURO.0265-20.2021)

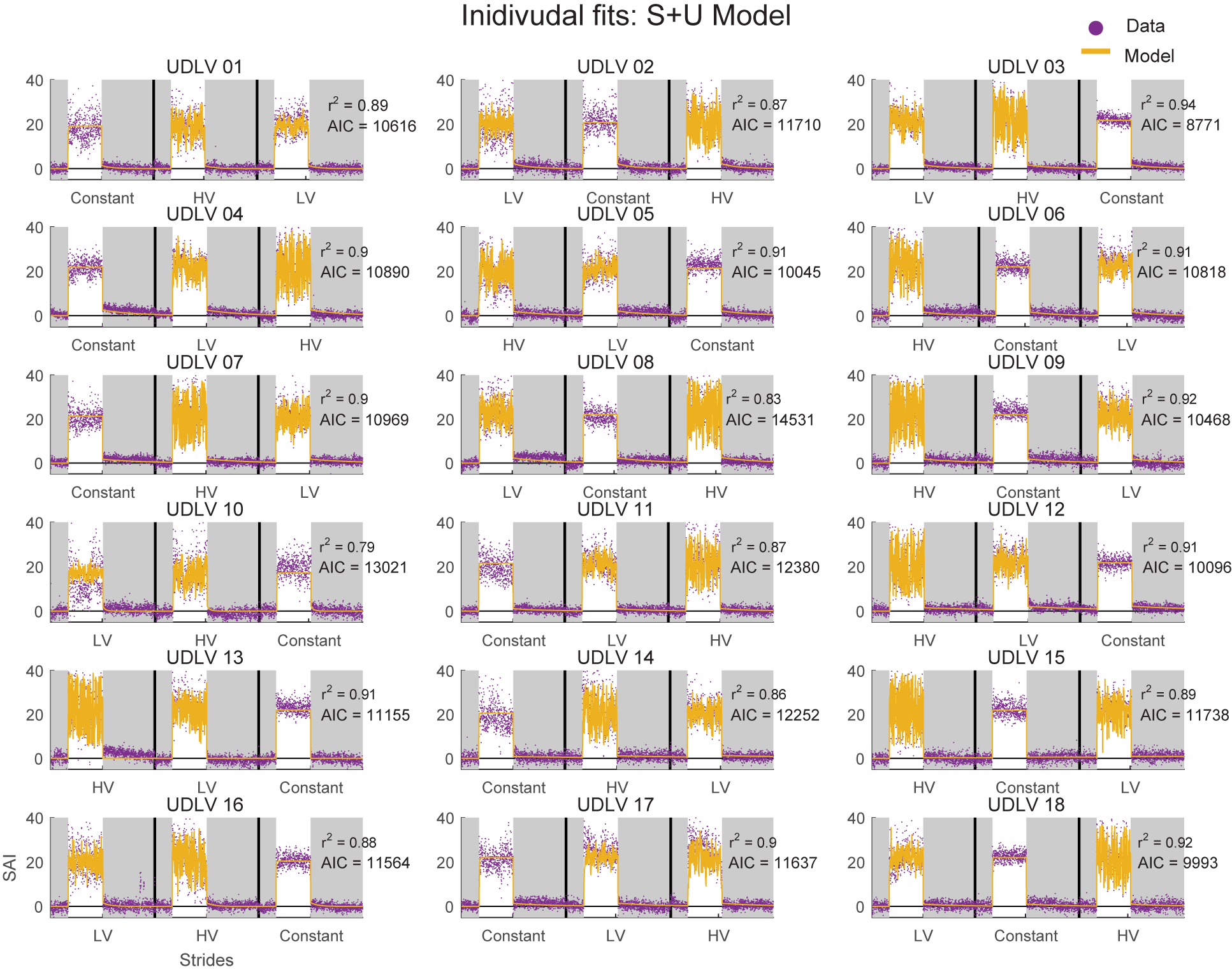

Supplement: Extended Data Figure 7-1 — Individual Strategy plus Use-Dependent model fits. The order in which each participant completed the conditions is on the x-axis. The r2 and AIC values for each fit are also provided. Download Figure 7-1, TIF file. [file enu-eN-NWRGR-0265-20-s02.tif]

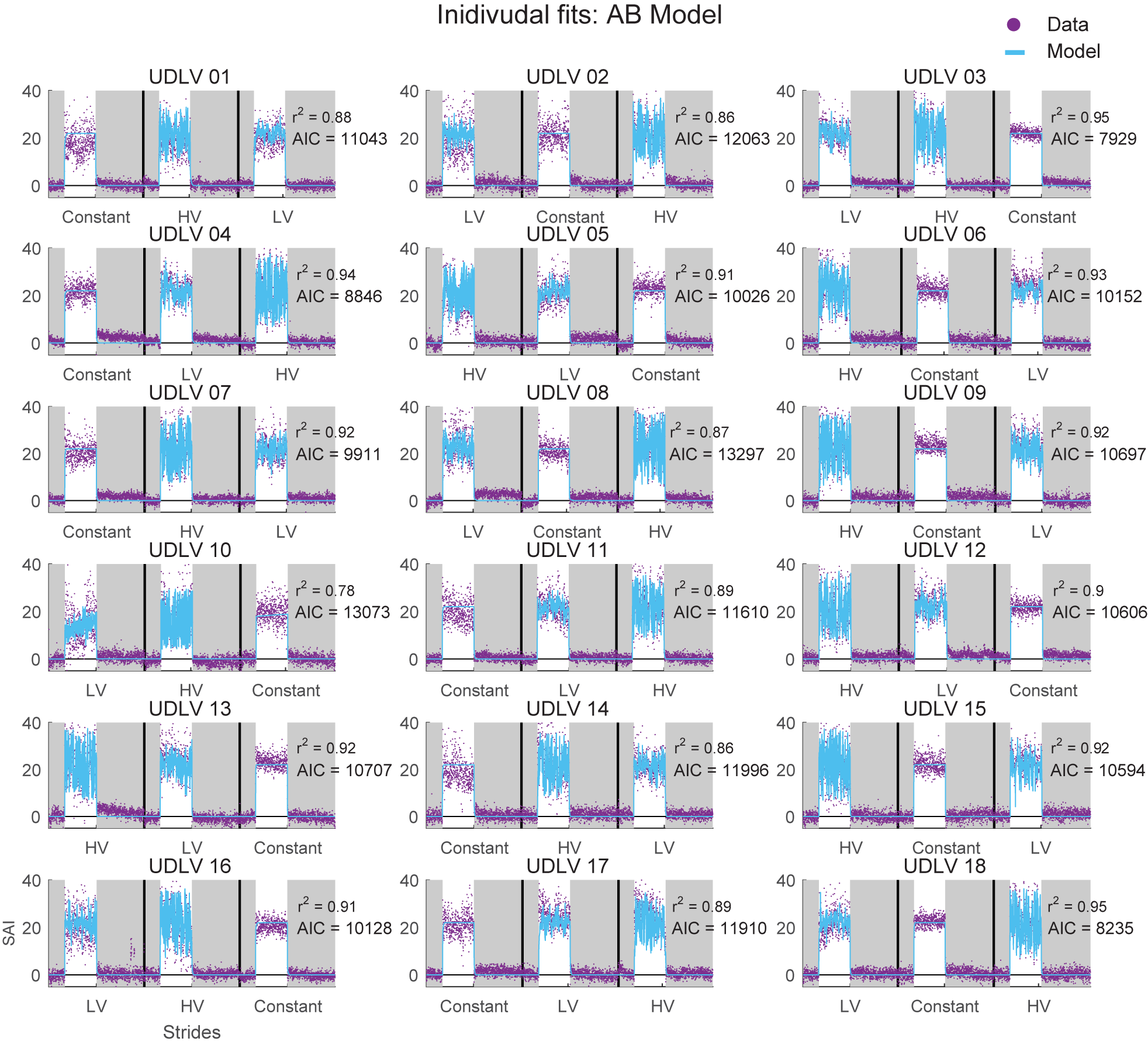

Supplement: Extended Data Figure 7-2 — Individual Adaptive Bayesian model fits. The order in which each participant completed the conditions is on the x-axis. The r2 and AIC values for each fit are also provided. Download Figure 7-2, TIF file. [file enu-eN-NWRGR-0265-20-s03.tif]

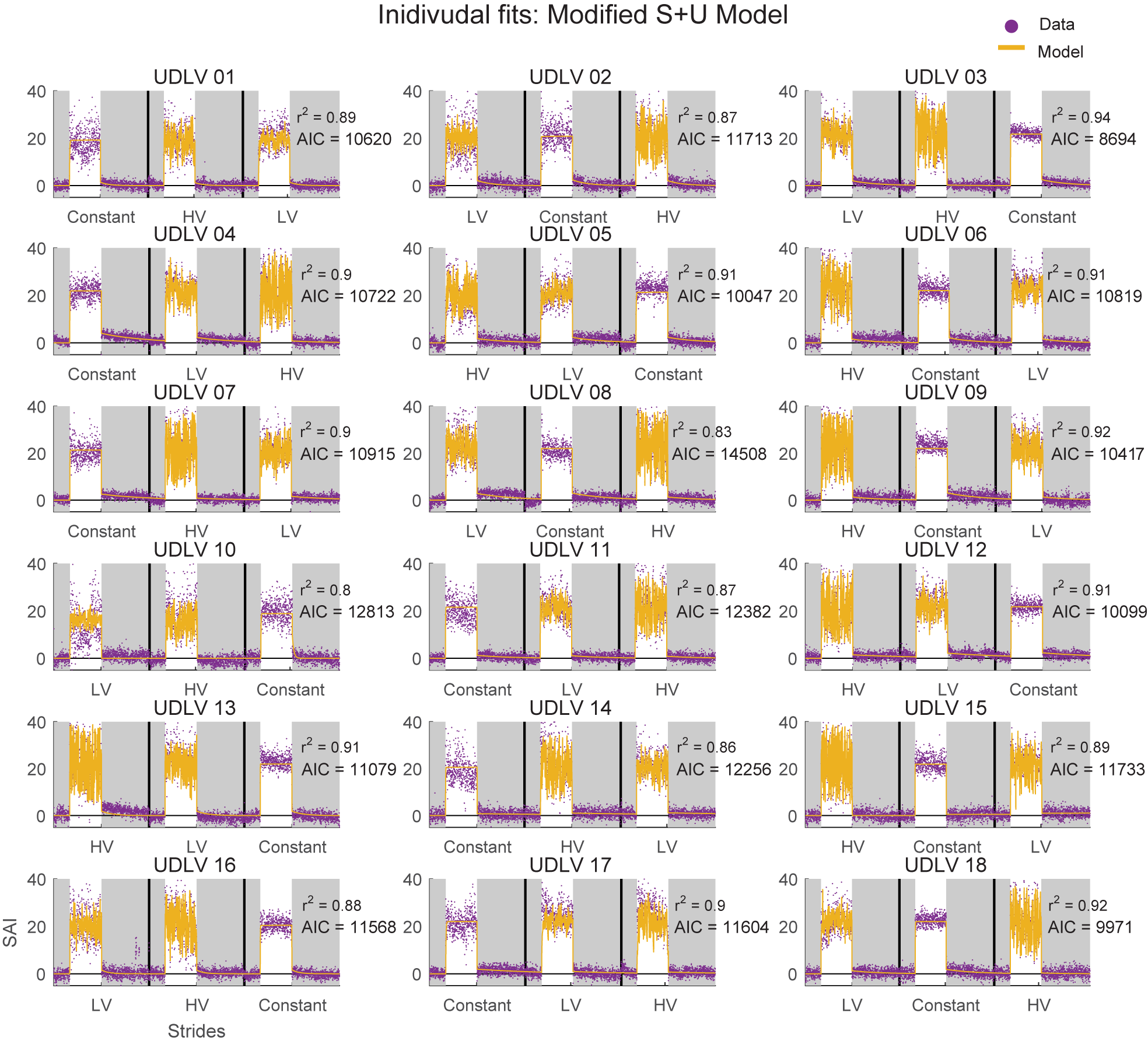

Supplement: Extended Data Figure 8-1 — Individual modified Strategy plus Use-Dependent model fits. The order in which each participant completed the conditions is on the x-axis. The r2 and AIC values for each fit are also provided. Download Figure 8-1, TIF file. [file enu-eN-NWRGR-0265-20-s05.tif]

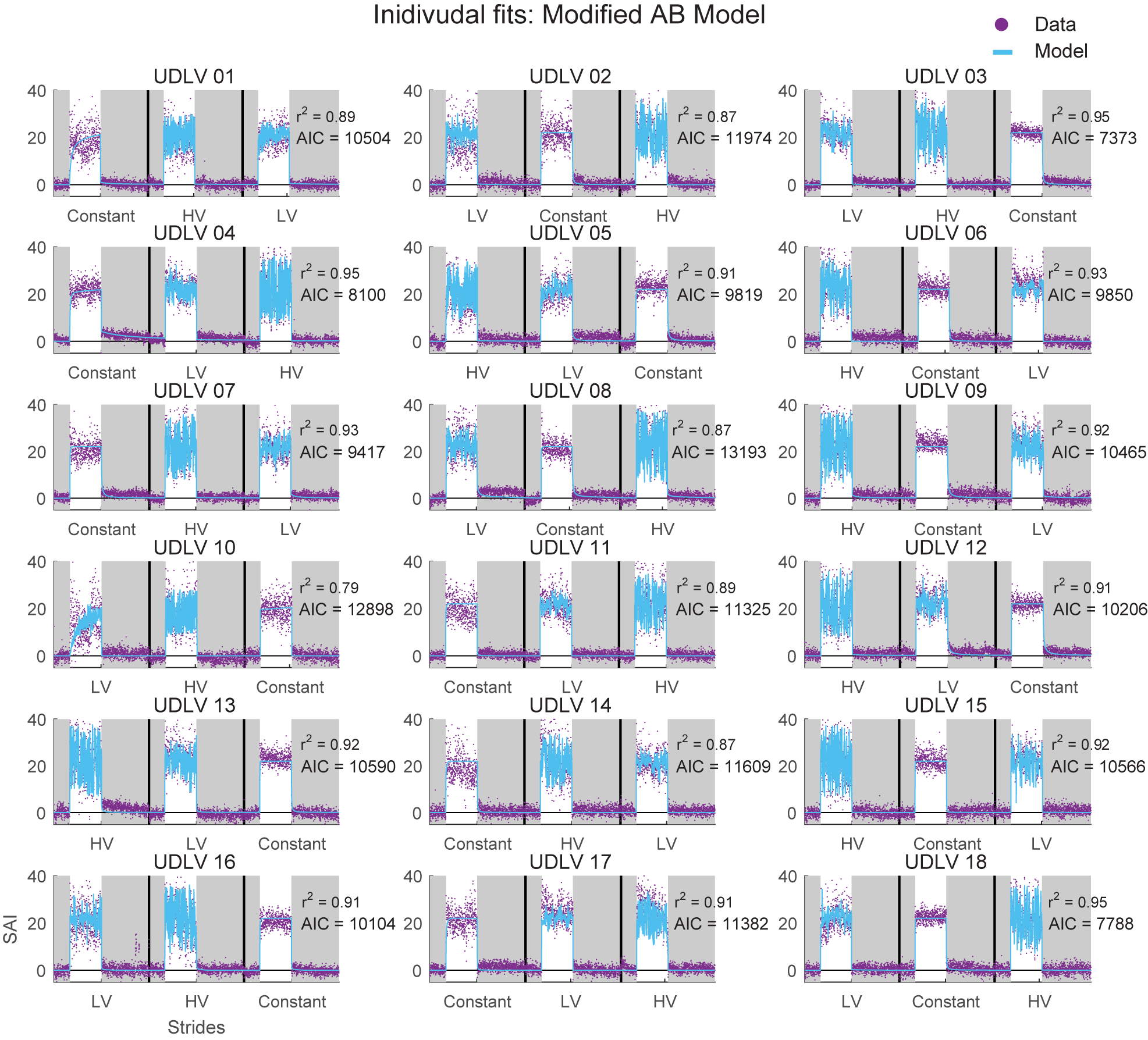

Supplement: Extended Data Figure 8-2 — Individual modified Adaptive Bayesian model fits. The order in which each participant completed the conditions is on the x-axis. The r2 and AIC values for each fit are also provided. Download Figure 8-2, TIF file. [file enu-eN-NWRGR-0265-20-s04.tif]
